# Novel polycyclic meroterpenoids with protein tyrosine phosphatase 1B inhibitory activity isolated from desert-derived fungi *Talaromyces* sp. HMT-8

**DOI:** 10.1007/s13659-025-00530-x

**Published:** 2025-07-18

**Authors:** Xin-yi Zhai, Jin-jie Liu, Cui-duan Wang, Yi-fan Dou, Jian-hua Lv, Li-an Wang, Jin-xiu Zhang, Zhuang Li

**Affiliations:** 1 College of Life Sciences, Hebei Normal University, Shijiazhuang, 050000 People’s Republic of China; 2 School of Land Science and Space Planning, Hebei GEO University, Shijiazhuang, 050000 People’s Republic of China

**Keywords:** *Talaromyces* sp., Polycyclic meroterpenoids, PTP1B, Enzyme kinetics, Molecular docking

## Abstract

Seven previously undescribed polycyclic meroterpenoids talarines K–Q (**1**–**7**), along with five known ones (**8**–**12**), were isolated from desert-derived fungi *Talaromyces* sp. HMT-8. The structure of the novel compounds were elucidated using spectroscopic methods, including electronic circular dichroism (ECD), HRESIMS and nuclear magnetic resonance (NMR) spectroscopy. Among the isolated meroterpenoids, compounds **3**, **5**, and **7** exhibited rare chlorine substitution patterns. Halogenation, particularly chlorination, is uncommon in natural meroterpenoids and implies the involvement of halogenase enzymes during biosynthesis. Compounds **1**–**12** were evaluated for their inhibitory activity against protein tyrosine phosphatase 1B (PTP1B). Compounds **1**–**4** and **12** exhibited inhibitory activity against PTP1B with IC₅₀ values ranging from 1.74 to 17.60 μM. Among them, compounds **2** and **12** displayed significant inhibitory effects with an IC₅₀ value of 1.74 and 3.03 μM, respectively. Furthermore, Molecular docking analysis revealed that compounds **2** and **12** bind tightly to the catalytic site of PTP1B, forming key hydrogen bonding and hydrophobic interactions. Enzyme kinetics studies further demonstrated that both compounds act as competitive inhibitor.

**Graphical Abstract:**

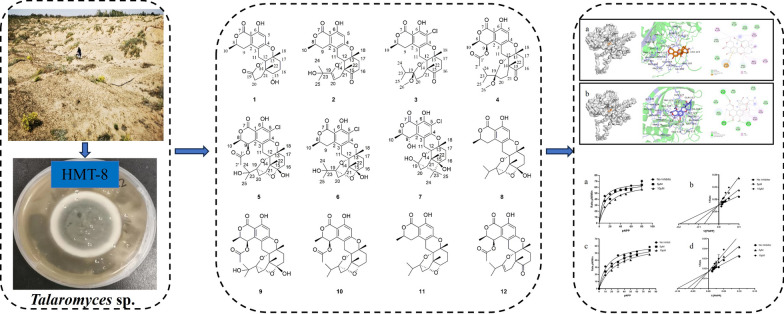

**Supplementary Information:**

The online version contains supplementary material available at 10.1007/s13659-025-00530-x.

## Introduction

**Fig. 1:**
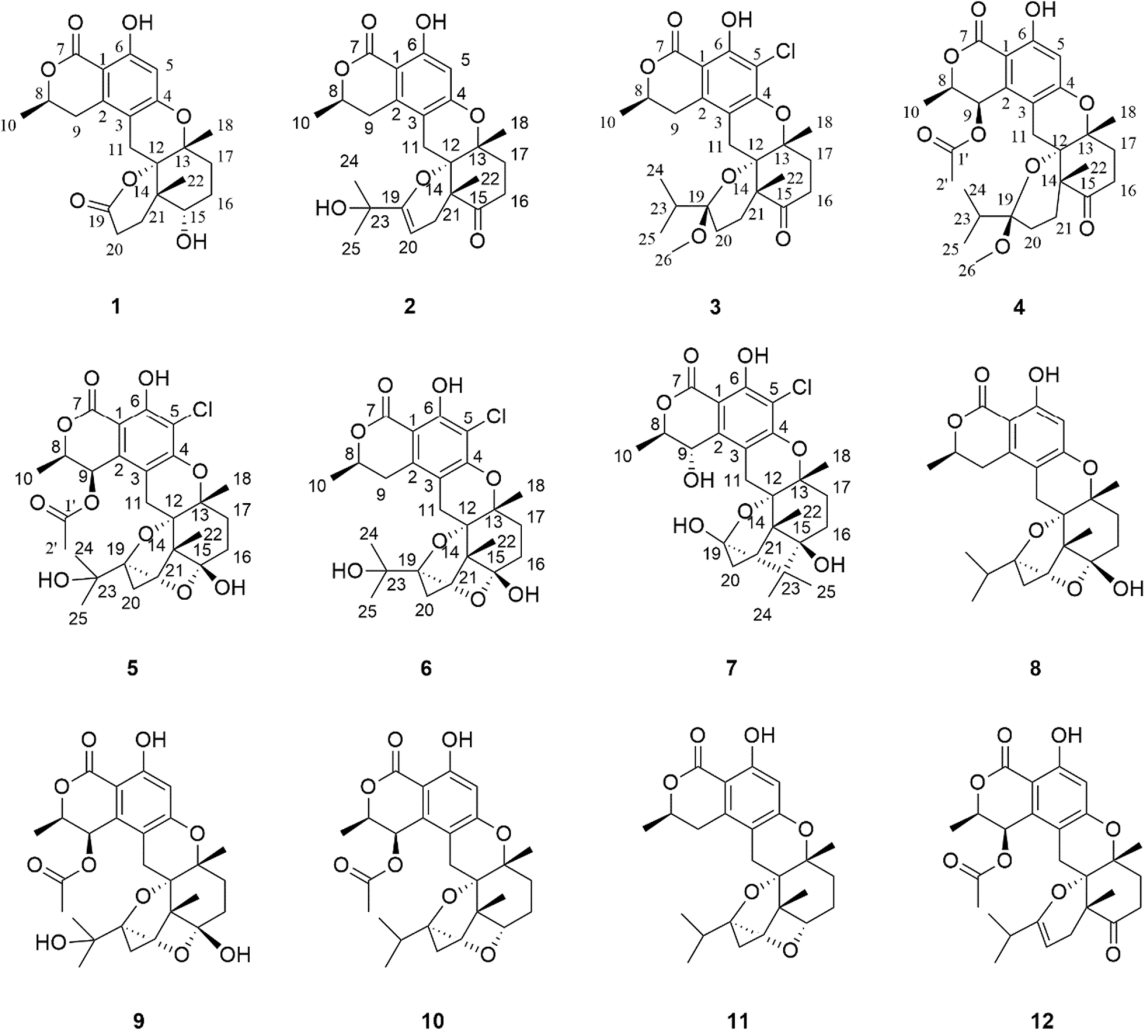
Chemical structures of **1**–**12** isolated from HMT-8

**Fig. 2:**
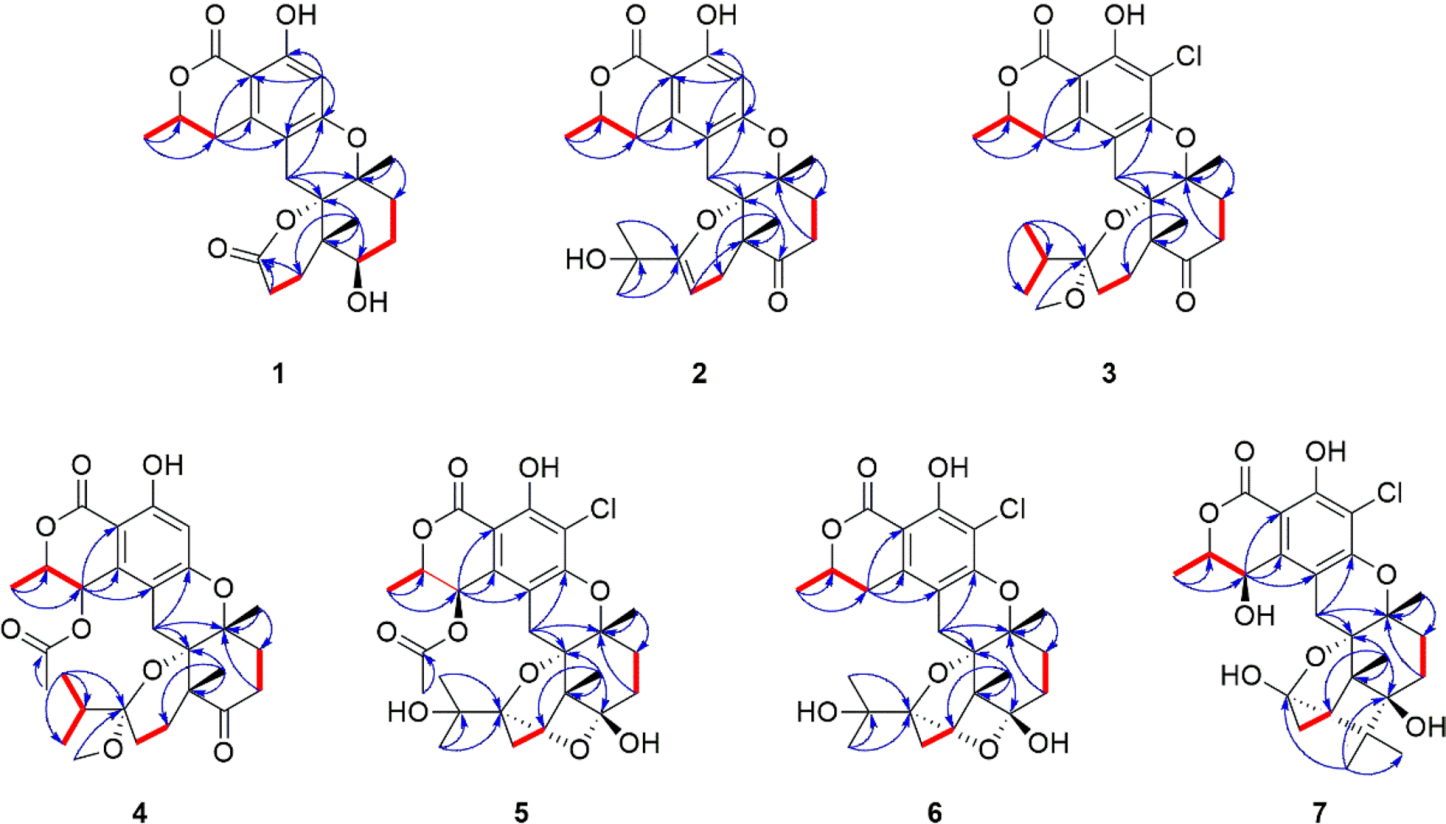
Key HMBC (blue arrow) and ^1^H-^1^H COSY (bold red line) correlations of **1**–**7**

**Fig. 3:**
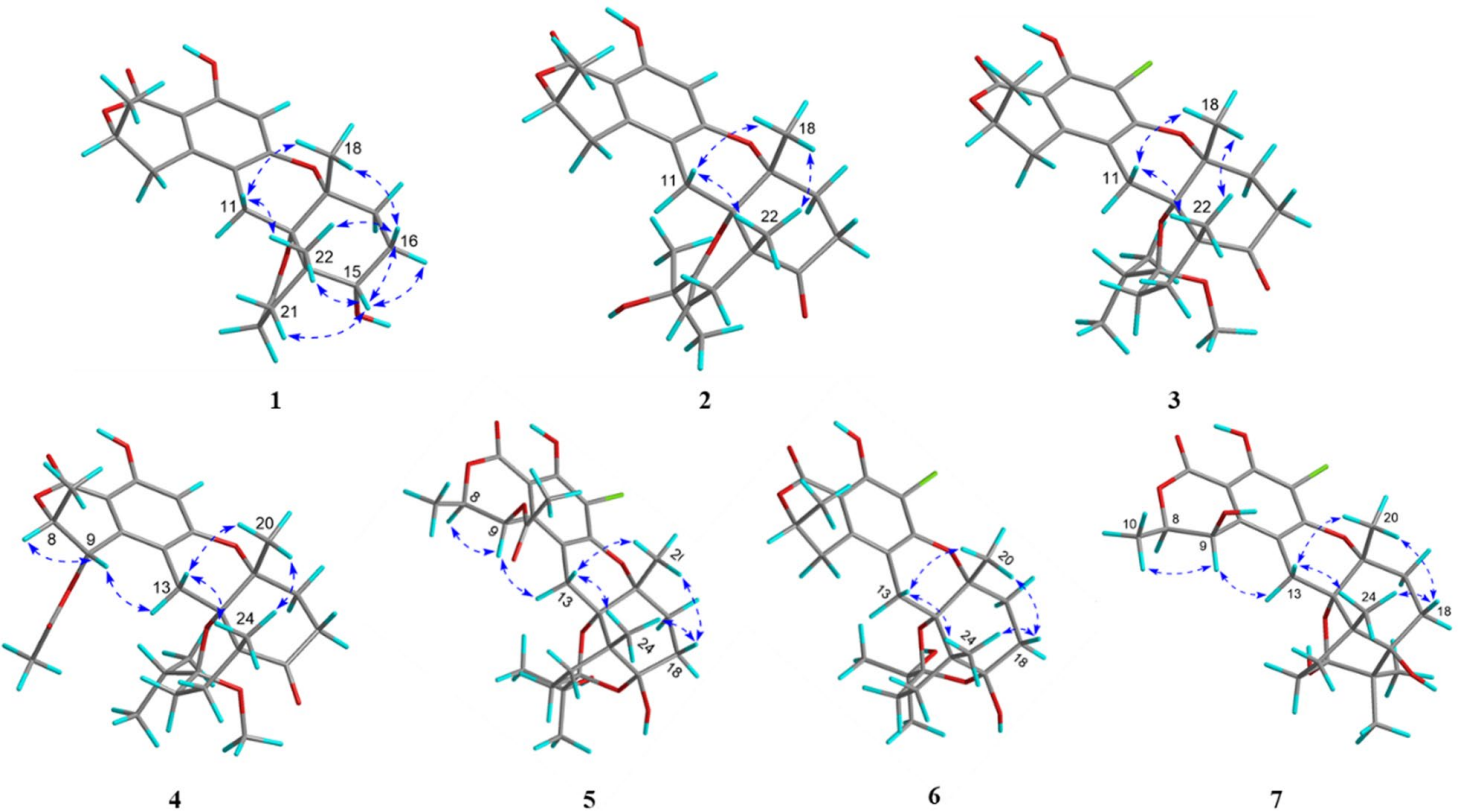
Key NOESY (double blue arrows) correlations of **1**–**7**

**Fig. 4:**
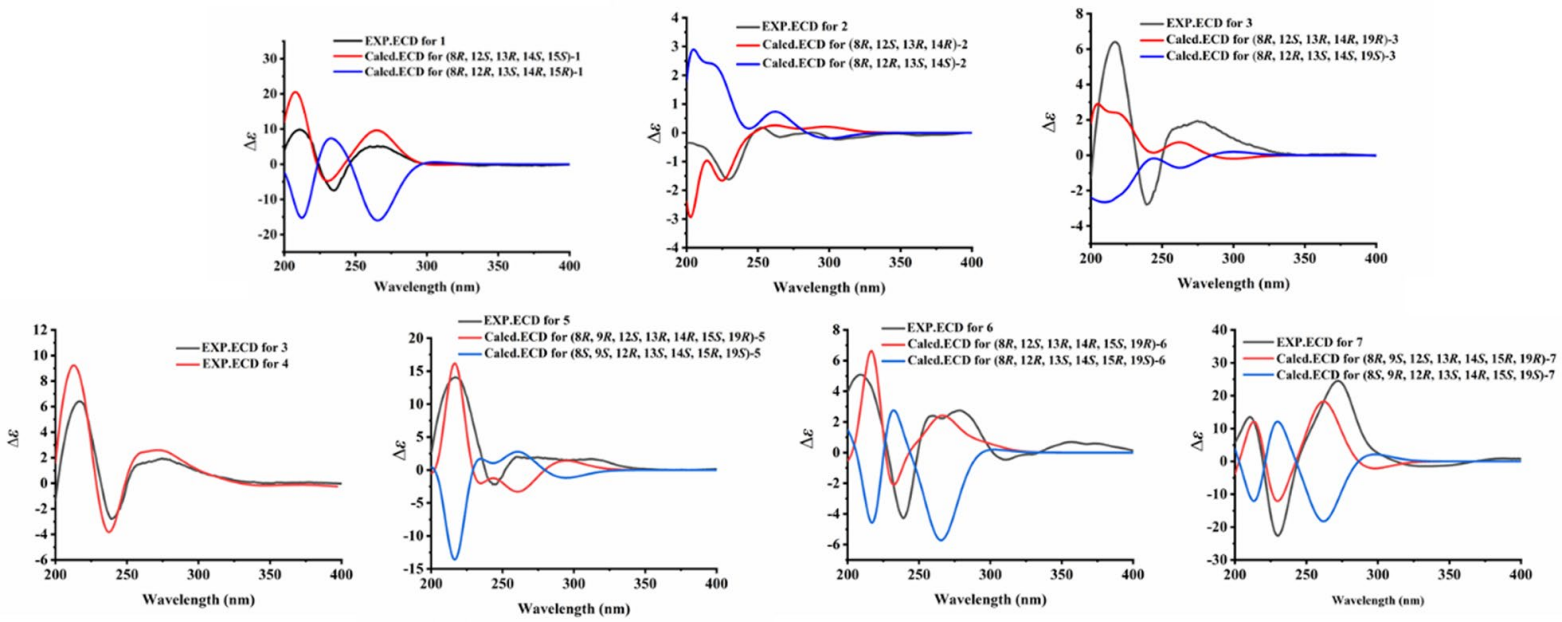
Comparison of experimental and calculated ECD spectra of **1**–**7**

**Fig. 5:**
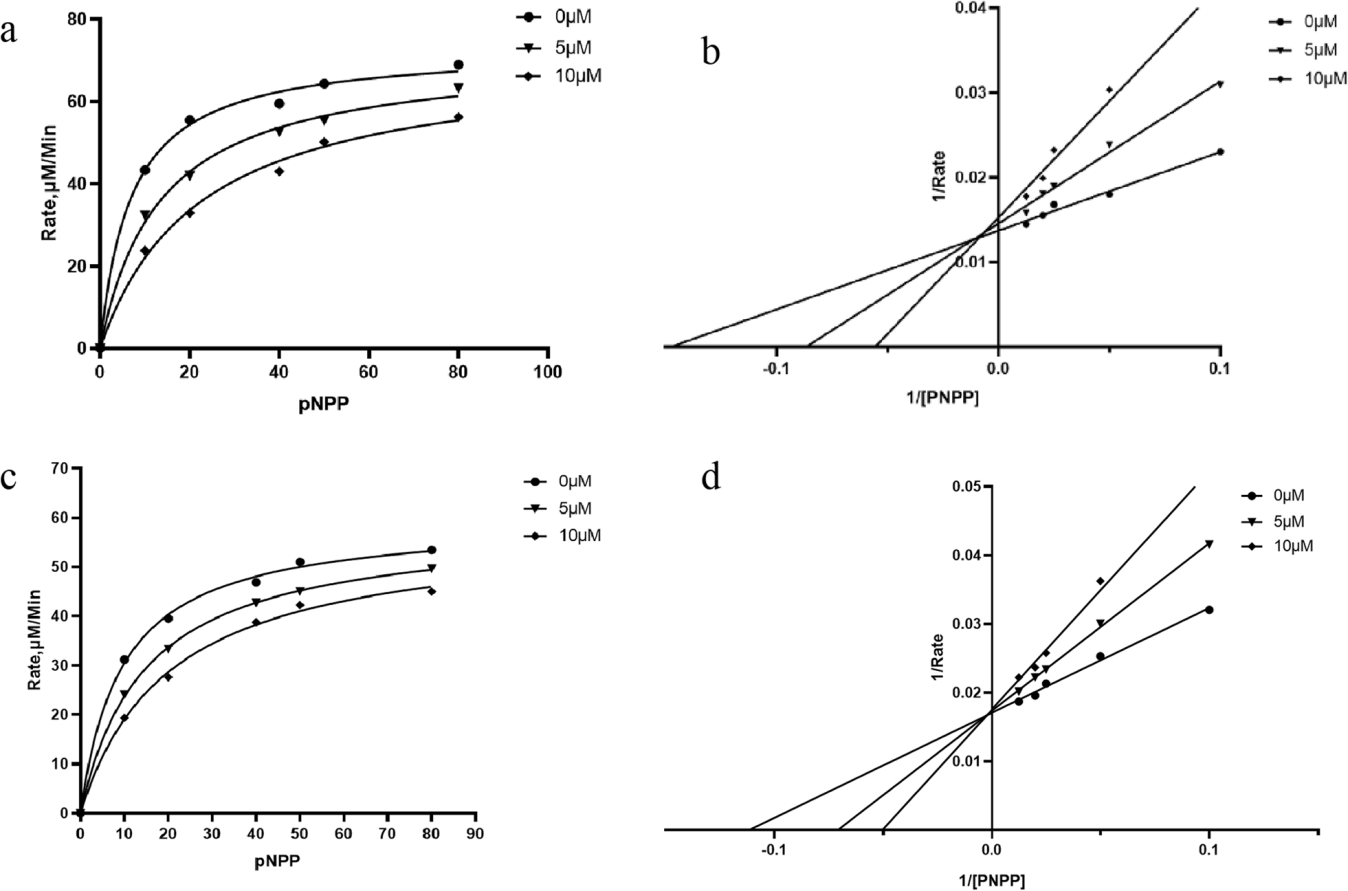
Kinetic analysis of the inhibition of PTP1B by **2** (**a**, **b**) and **12** (**c**, **d**)

**Fig. 6:**
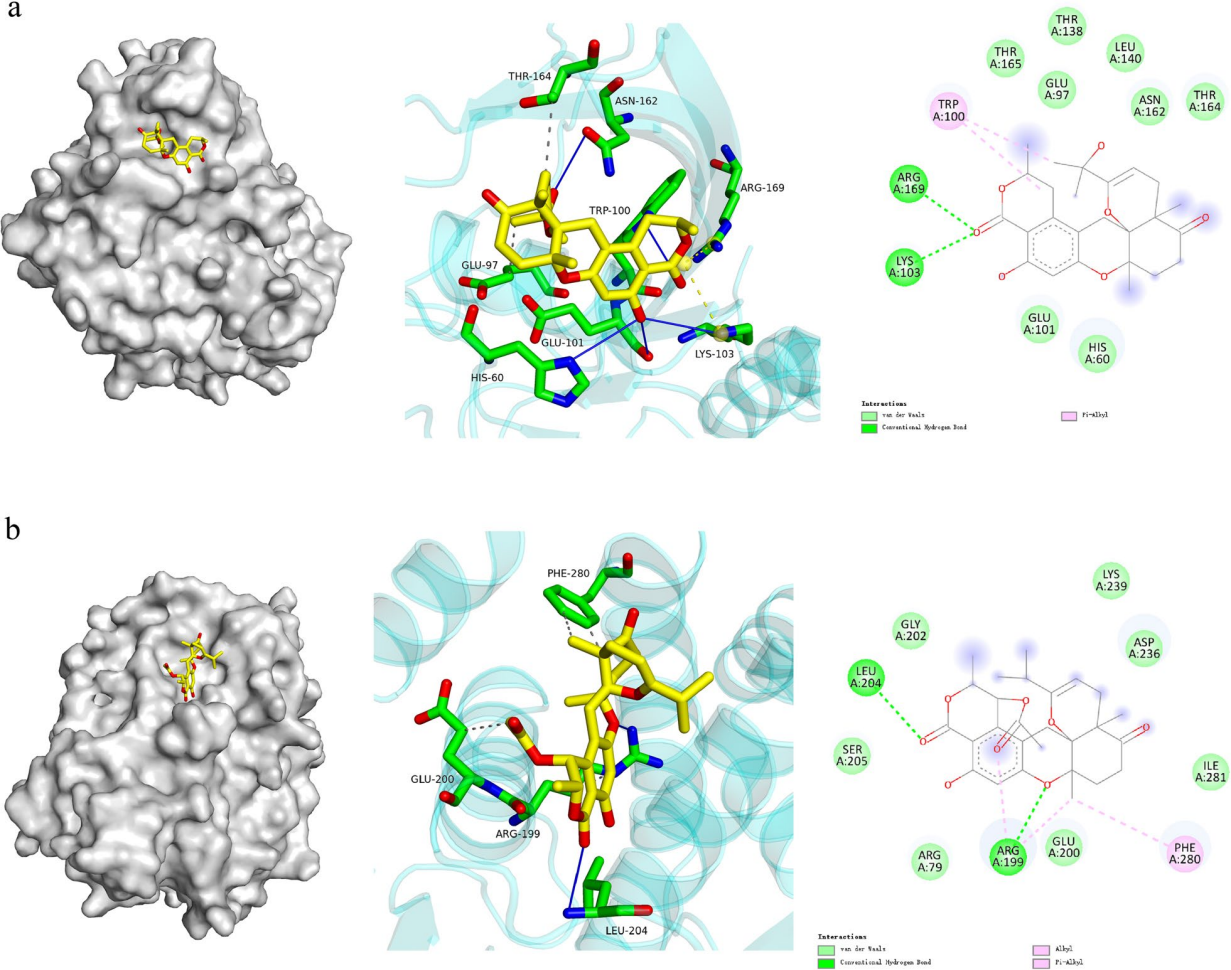
Molecular docking pictures of compounds **2** (**a**) and **12** (**b**)

The meroterpenoids are a highly diverse class of natural products. They are hybrid natural products, partially derived from the terpenoid biosynthetic pathway, exhibiting diverse core structures, among which some feature a C3-oxidized drimane skeleton [1, 6]. Recently, meroterpenoids have attracted attention due to their rich chemical diversity and biological activities. In the past decade, only 26 methoxysteroids, constructed from drimane-type sesquiterpenoid units and C10 polyketide moieties, have been reported [2–7]. Previous studies have indicated that *Talaromyces* sp. could produce secondary metabolites such as meroterpenoids, polyketides and furanosteroids with novel chemical structures. The meroterpenoids isolated from *Talaromyces* sp. contain polycyclic skeletons and exhibit significant in vitro enzyme inhibitory and antiviral activities [8, 9] (Figs. 1, 2, 3, 4, 5, 6).

Protein tyrosine phosphatase-1B (PTP1B) is a member of the protein tyrosine phosphatase (PTP) family and is localized in the endoplasmic reticulum of cells in tissues such as adipose and liver [10]. PTP1B plays an essential role across various physiological pathways, including cell proliferation, apoptosis, and differentiation [11]. The PTP1B functions by dephosphorylating insulin and leptin receptors, thereby inducing their inactivation [12, 13]. Inhibitors targeting this enzyme hold therapeutic promise for managing type 2 diabetes and obesity. Therefore, bioactive compounds with PTP1B inhibitory activity may exhibit enhanced therapeutic efficacy in the treatment of type 2 diabetes.

Finding bioactive compounds from natural products has become one of the important ways to develop innovative drugs. The extensive pharmacological activities and structural diversity of fungal natural products have attracted the attention of researchers [14]. We have been dedicated to discovering novel bioactive compounds from fungi. In this work, a desert-derived fungi *Talaromyces* sp. HMT-8 was isolated from soil collected in Alashan region in the west of Inner Mongolia Autonomous Region of China. The chemical investigation on HMT-8 led to the identification of seven novel polycyclic meroterpenoids talarines K–Q (**1**–**7**) and five known ones (**8**–**12**). All the isolated compounds were tested for their inhibitory activities against PTP1B. Many of these meroterpenoids exhibited inhibitory activities. This paper reports on the isolation, structural elucidation and PTP1B inhibitory activity of polycyclic meroterpenoids extracted from *Talaromyces* sp. HMT-8.

## Results and discussion

### Structure elucidation

Compound **1** displayed the molecular formula, C_22_H_26_O_7_, by analysis of its HREISMS data ([M + H]^+^ *m/z* 403.1748, calcd for C_22_H_27_O_7_, 403.1757), indicating 10 degrees of unsaturation. The ^1^H NMR data (Table 2) of **1 **showed resonances for one aromatic proton at *δ*_H_ 6.24 (1H, s, H-5), two oxygenated methine protons at *δ*_H_ 4.66 (1H, m, H-8) and 3.67 (1H, t, *J* = 3.0 Hz, H-15), and three methyl groups at *δ*_H_ 1.51 (3H, d, *J* = 6.5 Hz, H_3_-10), 1.40 (3H, s, H_3_-18), and 1.04 (3H, s, H_3_-22). The ^13^C NMR data (Table 1) of **1** showed a total of 22 carbon signals, including two ester carbonyls at *δ*_C_ 176.7 (C-19) and 171.9 (C-7), six aromatic carbons assignable to a benzene ring, six methylenes, two methines (including one oxygenated), three non-protonated carbons (including two oxygenated), and three methyls. The planar structure of **1** was further elucidated by 2D-NMR spectroscopic analysis. The ^1^H−^1^H COSY correlations (Fig. 2) of H_3_-10/H-8/H-9 together with the HMBC correlations (Fig. 2) from H-9 to C-2, C-3, and C-11, and from H-5 to C-1, C-3, C-4, and C-6 confirmed the presence of the isochromanone moiety. The HMBC correlations from H_2_-11 to C-4, C-12, and C-13, from H_3_-18 to C-13 and C-17, from H_3_-22 to C-12, C-14, C-15, and C-21, and from H_2_-20 to C-19, along with the ^1^H − ^1^H COSY correlations of H_2_-17/H_2_-16/H-15 and H_2_-10/H_2_-21 established the remaining nor-sesquiterpene moiety. Therefore, the planar structure of **1** was determined, as depicted in Fig. 1.

**Table 1:** The ^13^C NMR (150 MHz, CD_3_OD) spectroscopic data for compounds **1**–**7**

NO	1	2	3	4	5	6	7
1	102.9, C	102.8, C	103.3, C	103.2, C	103.2, C	103.0, C	102.3, C
2	142.1, C	141.7, C	139.0, C	138.1, C	135.9, C	139.3, C	139.9, C
3	111.4, C	109.7, C	112.6, C	113.5, C	113.2, C	111.8, C	112.8, C
4	161.3, C	161.0, C	156.6, C	161.5, C	157.2, C	156.9, C	157.2, C
5	103.8, CH	103.4, CH	108.5, C	106.1, CH	111.2, C	108.5, C	110.0, C
6	163.3, C	163.5, C	158.7, C	163.3, C	158.8, C	158.7, C	158.6, C
7	171.9, C	171.7, C	171.6, C	170.6, C	170.6, C	171.7, C	171.4, C
8	76.6, CH	76.5, CH	76.7, CH	65.6, CH	77.8, CH	76.8, CH	79.4, CH
9	32.0, CH_2_	31.9, CH	31.8, CH_2_	77.5, CH	65.4, CH	31.6, CH	63.9, CH
10	21.0, CH_3_	20.9, CH_3_	21.0, CH_3_	16.6, CH_3_	16.5, CH_3_	20.9, CH_3_	16.5, CH_3_
11	28.2, CH_2_	22.6, CH_2_	26.3, CH_2_	26.2, CH_2_	28.1, CH_2_	28.4, CH_2_	28.2, CH_2_
12	77.1, C	81.0, C	79.1, C	78.9, C	78.5, C	78.8, C	79.4, C
13	81.0, C	79.1, C	81.5, C	80.7, C	81.9, C	81.5, C	82.3, C
14	43.3, C	50.4, C	51.1, C	51.0, C	38.3, C	38.3, C	41.5, C
15	72.9, CH	211.1, C	214.3, C	214.2, C	99.8, C	99.9, C	76.5, C
16	27.2, CH_2_	34.7, CH_2_	36.0, CH_2_	36.1, CH_2_	32.1, CH_2_	32.1, CH_2_	30.4, CH_2_
17	28.6, CH_2_	34.5, CH_2_	32.7, CH_2_	32.8, CH_2_	32.8, CH_2_	32.9, CH_2_	35.1, CH_2_
18	24.7, CH_3_	23.5, CH_3_	23.4, CH_3_	23.3, CH_3_	24.0, CH_3_	23.9, CH_3_	24.5, CH_3_
19	176.7, C	156.6, C	104.4, C	104.4, C	100.5, C	100.5, C	100.9, C
20	29.3, CH_2_	93.4, CH	24.8, CH_2_	24.7, CH_2_	25.3, CH_2_	25.4, CH_2_	30.8, CH_2_
21	29.2, CH_2_	28.7, CH_2_	26.6, CH_2_	26.7, CH_2_	23.7, CH_2_	23.7, CH_2_	26.6, CH_2_
22	19.8, CH_3_	24.3, CH_3_	25.6, CH_3_	25.6, CH_3_	19.2, CH_3_	19.2, CH_3_	19.6, CH_3_
23		71.5, C	33.5, CH	33.5, CH	74.5, C	74.6, C	48.3, C
24		27.8, CH_3_	18.0, CH_3_	16.5, CH_3_	24.8, CH_3_	24.8, CH_3_	25.0, CH_3_
25		27.9, CH_3_	16.5, CH_3_	17.9, CH_3_	24.6, CH_3_	24.5, CH_3_	18.7, CH_3_
26			48.7, CH_3_	49.0, CH_3_			
1’				171.7, C	171.8, C		
2’				20.4, CH_3_	20.4, CH_3_		

The relative configuration of **1** was determined by NOESY experiment. The NOESY cross peaks (Fig. 3) of H_3_-22/H-16*β* (*δ*_H_ 1.97), H_3_-18/H-16*β*, H_3_-18/H-11*β* (*δ*_H_ 2.68), H_3_-22/H-11*β*, and H_3_-22/H-15 indicated that H-15, H_3_-18, and H_3_-22 were *β*-orientations; simultaneously, C-12 was assigned as S*-configuration. The absolute configuration at C-8 was proposed as *R* based on the biosynthetic origin [5, 15]. The calculated electronic circular dichroism (ECD) curve (Fig. 4) for compound 1 corresponded well with the experimental ECD curve, indicating its absolute configuration as 8*R*,12*S*,13*R*,14*S*,15*S*. Therefore, compound **1** was named talarine K.

Compound **2** possessed a molecular formula C_25_H_30_O_7_, as determined by the HRESIMS data ([M + H]^+^ *m/z* 443.2061, calcd for C_25_H_31_O_7_, 443.2070). The ^1^H NMR spectrum of **2** displayed characteristic signals including one oxygen-bearing methine proton at *δ*_H_ 4.57 (1H, m, H-8), and five distinct methyl signals (four singlets and one doublets). The ^13^C NMR spectra of **2** showed 25 carbon resonances, including two carbonyl carbons (one ester carbonyl at *δ*_C_ 171.7 and one keto carbonyl *δ*_C_ 211.1), eight olefinic carbons containing a benzene ring, four nonprotonated carbons at *δ*_C_ 81.0 (C-12), 79.1 (C-13), 71.5 (C-23), and 50.4 (C-14), five methylenes at *δ*_C_ 34.7 (C-16), 34.5 (C-17), 31.9 (C-9), 28.7 (C-21), and 22.6 (C-11), one oxygenated methine at *δ*_C_ 76.5 (C-8), and five methyls at *δ*_C_ 27.9 (C-25), 27.8 (C-24), 24.3 (C-22), 23.5 (C-18), and 20.9 (C-10). The NMR data (Tables 1 and 2) of **2** closely resembled those of talaromyolide I [6], with the main difference being the presence of an oxygenated non-protonated carbon (C-23) and the absence of an oxygenated methine group (C-9), as supported by the HMBC correlations (Fig. 2) from H_3_-25 to C-23 and the ^1^H−^1^H COSY correlations (Fig. 2) of H_3_-10/H-8/H_2_-9. The cross-peaks of H_3_-18/H_3_-22/H-11*β* (*δ*_H_ 2.84) established the relative configuration of **2**. The absolute configuration of compound **2** was determined by comparing its experimental ECD spectrum with the calculated spectra (Fig. 4), confirming the assignment as 8*R*, 12*S*, 13*R*, 14*R*. Accordingly, it was named talarine L.

**Table 2:** The ^1^H NMR (600 MHz, CD_3_OD, *J* in Hz)) spectroscopic data for compounds **1**–**7**

NO	1	2	3	4	5	6	7
1	–	–	–	–	–	–	–
2	–	–	–	–	–	–	–
3	–	–	–	–	–	–	–
4	–	–	–	–	–	–	–
5	6.24, s	6.29, s	–	6.46, s	–	–	–
6	–	–	–	–	–	–	–
7	–	–	–	–	–	–	–
8	4.66, m	4.57, m	4.72, m	4.85–4.79, m	4.84, m	4.70, m	4.62, qd, (6.7, 1.8)
9	3.07, dd, (16.9, 3.4)	2.97, overlapped	3.11, dd, (17.2, 3.6)	6.19, d, (2.0)	6.25, d, (1.9)	3.14, dd, (16.9, 3.5)	4.70, d, (2.0)
	2.63, m	2.70, overlapped	2.75, dd, (17.0, 11.3)	–	–	2.67, dd, (17.0, 11.3)	
10	1.51, d, (6.5)	1.50, d, (6.3)	1.55, d, (6.2)	1.47, d, (6.5)	1.45, d, (6.5)	1.52, d, (6.2)	1.57 (3H, d, *J* = 6.6 Hz)
11	2.68, m	2.84, d, (17.1)	3.64, d, (16.7)	3.72, d, (17.1)	3.02, d, (16.8)	2.92, d, (16.5)	3.13, d, (16.9)
		2.79, d, (17.1)	2.85, d, (16.7)	2.76, d, (17.1)	2.90, d, (16.8)	2.81, d, (16.5)	3.06, d, (16.9)
12	–	–	–	–	–	–	–
13	–	–	–	–	–	–	–
14	–	–	–	–	–	–	
15	3.67, t, (3.0)	–	–	–	–	–	
16	1.97, m	2.97, overlapped	2.85, overlapped	2.83, td, (15.0, 6.6)	1.97, overlapped	1.97, overlapped	1.97, overlapped
	1.86, m	2.34, m	2.44, overlapped	2.44–2.38, m	1.77, overlapped	1.77, overlapped	1.81, overlapped
17	2.35, m	2.54, td, (13.6, 5.4)	2.72, overlapped	2.65, td, (13.8, 5.0)	2.57, td, (13.2, 5.1)	2.57, td, (13.0, 5.3)	2.60, m
	1.60, m	2.12, ddd, (12.7, 6.8, 2.1)	2.04, overlapped	1.99–1.93, m	1.77, overlapped	1.77, overlapped	1.82, overlapped
18	1.40, s	1.67, s	1.56, s	1.52, s	1.32, s	1.34, s	1.35, s
19	–	–	–		–	–	–
20	2.55, overlapped	4.91, overlapped	1.77, td, (13.7, 5.7)	1.75, td, (13.8, 5.4)	2.03, overlapped	2.02, overlapped	1.97, overlapped
	1.70, m		1.37, m	1.42–1.36, m	1.90, overlapped	1.92, overlapped	1.78, overlapped
21	2.55, overlapped	2.66, overlapped	2.00, overlapped	2.06–2.00, m	2.03, overlapped	2.02, overlapped	2.09, m
	2.41, m	1.95, dd, (17.7, 2.2)	1.93, overlapped	1.88, td, (13.7, 4.7)	1.90, overlapped	1.92, overlapped	1.77, overlapped
22	1.04, s	1.32, s	1.26, s	1.19, s	1.08, s	1.13, s	1.02, s
23		–	1.97, overlapped	1.98–1.92, m	–	–	–
24		1.04, s	0.78, d, (6.7)	0.70, d, (6.9)	1.17, s	1.18, s	1.06, s
25		0.67, s	0.70, d, (6.9)	0.76, d, (6.7)	1.12, s	1.13, s	1.19, s
26			2.57, s	2.59, s			
1’				–	–		
2’				2.13, s	2.12, s		

Compound **3** was purified as a colorless oil, and its HRESIMS analysis ([M − H]^−^ *m/z* 491.1837, calcd for C_26_H_32_ClO_7_, 491.1843) indicated a molecular formula C_26_H_33_ClO_7_. The ^1^H NMR spectral data (Table 2) of **3** showed one oxygenated methine proton at *δ*_H_ 4.72 (1H, m, H-8), five methyl signals (two singlets and three doublets), as well as a methoxyl signal at *δ*_H_ 2.57 (3H, s, H_3_-26). The ^13^C NMR spectrum (Table 1) revealed the presence of 26 carbon signals, including two carbonyl carbons (one ester carbonyl and one keto carbonyl), six aromatic carbons, one ketal carbon at *δ*_C_ 104.4 (C-19), one oxygenated methine at *δ*_C_ 76.7 (C-8), six methylenes, three nonprotonated carbons containing two oxygenated at *δ*_C_ 81.5 (C-13) and 79.1 (C-12), and six methyls including an oxygenated at *δ*_C_ 48.7 (C-26). Detailed analyses of the above NMR data suggested that the structure of **3** was similar to that of **2**, with the main difference being the presence of a methoxyl group (C-26), a methine group (C-23), and a methylene group (C-20), coupled with the absence of two olefinic carbons and the singlet H-5 proton signal, as supported by the HMBC correlations (Fig. 2) from H_3_-24 to C-19, C-23, and C-25, and the ^1^H−^1^H COSY correlations (Fig. 2) of H_3_-24/H-23/H_3_-25, combined with the molecular formula. Finally, the relative configuration of **3** was determined by the cross peaks of H_3_-18/H-11*β* (*δ*_H_ 2.85)/H_3_-22. To determine the configuration at C-19, DFT calculations of ^1^H and ^13^C NMR chemical shifts were performed for four possible diastereomers (see SI). Subsequent DP4 + analysis (Fig. S50) revealed that the C-19 stereocenter as *R*. The absolute configuration of **3** was determined from ECD spectrum in comparison to calculated spectra (Fig. 4), confirming the absolute configuration of (8*R*,12*S*,13*R*,14*R*,19*R*)-**3**. Therefore, compound **3** was named talarine M.

Compound **4** had a molecular formula C_28_H_36_O_9_, as disclosed by the HRESIMS spectrum ([M + H]^+^ *m/z* 517.2438, calcd for C_28_H_37_O_9_, 517.2435). The ^1^H NMR spectral data (Table 2) of **4** showed two oxygen-bearing methine protons at *δ*_H_ 6.19 (1H, d, *J* = 2.0 Hz, H-9) and 4.85−4.79 (1H, m, H-8), an aromatic proton signal at *δ*_H_ 6.46 (1H, s, H-5), five methyl signals (two singlets and three doublets), as well as a methoxyl signal at *δ*_H_ 2.59 (3H, s). The ^13^C NMR spectrum (Table 1) showed a total of 28 carbon signals, including three carbonyl carbons (two ester carbonyls and one keto carbonyl), six aromatic carbons, one ketal carbon at *δ*_C_ 104.4 (C-19), two oxygen-bearing methines at *δ*_C_ 77.5 (C-9) and 65.6 (C-8), five methylenes at *δ*_C_ 36.1 (C-16), 32.8 (C-17), 26.7 (C-21), 26.2 (C-11), and 24.7 (C-20), three nonprotonated carbons containing two oxygenated at *δ*_C_ 80.7 (C-13) and 78.9 (C-12), and six methyls including an oxygenated at *δ*_C_ 49.0 (C-26). These ^1^H and ^13^C NMR data closely resembled those of **3**, suggesting that their structures were closely related. The key structural distinction was characterized by the presence of an acetyl group, an oxygenated methine group, and the H-5 singlet proton signal, concomitant with the absence of both a methylene group and a non-protonated aromatic carbon in **4**. The HMBC correlations (Fig. 2) from H_3_-2′ to C-1′, and from H-9 to C-1′, as well as the ^1^H − ^1^H COSY correlations (Fig. 2) of H_3_-10/H-8/H-9, confirmed that the C-9 methylene group in **3** was replaced by an acetyl group in **4**. Therefore, the planar structure of** 4** was assigned, and its relative configuration was determined according to the NOESY correlations (Fig. 3) of H_3_-18/H-11*β* (*δ*_H_ 2.78)/H_3_-22, H-9/H-11*α* (*δ*_H_ 3.72), and H-9/H-8. A strong NOE correlation is observed between H-9 and H-8, along with a weak correlation between H-9 and H-10, implying that H-9 and H-8 reside on the same face of the six-membered ring. The configuration at C-19 was established by DP4 + probability analysis based on calculated ^1^H and ^13^C NMR chemical shifts of four possible diastereomers, which indicated that structure 4–2 (see SI) best matched the experimental data with 100% probability, supporting the assignment of the C-19 stereocenter as *R*. The similar ECD spectra of indicated that compounds **3** and **4** share absolute configurations, so confirming the absolute configuration of (8*R*,12*S*,13*R*,14*R*,19*R*)-**4**. Therefore, compound **4** was named talarine N.

Compound **5** was isolated as a colorless oil, whose molecular formula was established as C_27_H_33_ClO_10_ by the HRESIMS data ([M + H]^+^ *m/z* 553.1841, calcd for C_27_H_34_O_10_Cl, 553.1829), bearing one more chlorine substituent than talaromyolide H [7]. The ^1^H NMR data (Table 2) of **5** showed two oxygen-bearing methine protons at *δ*_H_ 6.25 (1H, d, *J* = 1.9 Hz, H-9) and 4.84 (1H, m, H-8), one acetyl signal at *δ*_H_ 2.12 (3H, s, H-2’), and five methyl signals (four singlets and one doublet). The ^13^C NMR data of **5** (Table 1) exhibited 27 carbon resonances, including two ester carbonyl carbons at *δ*_C_ 171.8 (C-1’) and 170.6 (C-7), six aromatic carbons assignable to a benzene ring, two ketal carbons at *δ*_C_ 100.5 (C-19) and 99.8 (C-15), two oxygen-bearing methines at *δ*_C_ 77.8 (C-8) and 65.4 (C-9), five methylenes at *δ*_C_ 32.8 (C-17), 32.1 (C-16), 28.1 (C-11), 25.3 (C-20), and 23.7 (C-21), four nonprotonated carbons including three oxygenated at *δ*_C_ 81.9 (C-13), 78.5 (C-12), and 74.5 (C-23), and six methyls at *δ*_C_ 24.8 (C-24), 24.6 (C-25), 24.0 (C-18), 20.4 (C-2’), 19.2 (C-22), and 16.5 (C-10). These data suggested that **5** was a polycyclic meroterpenoid possessing a seco-drimane unit and an isocoumarin core structure, similar to that of talaromyolide H. The main difference between them lied in the disappearance of the singlet proton signal. HMBC correlations from H-9 to C-1, C-2, and C-3, and from H_2_-11 to C-4, C-12, and C-13, along with the ^1^H−^1^H COSY correlations of H_3_-10/H-8/H-9 were observed. The above data, with the aid of a molecular formula that showed the presence of a Cl atom, assigned **5** as 5-chlorotalaromyolide H. The relative configuration of **5** was determined to be the same as that of talaromyolide H based on the key NOESY correlations (Fig. 3) of H_3_-18/H-11*β* (*δ*_H_ 2.90), H_3_-22/H-11*β*, H_3_-18/H-16*β* (*δ*_H_ 1.97), H_3_-22/H-16*β*, and H-9/H-11*α* (*δ*_H_ 3.02). A strong NOE between H-9 and H-8, together with a weaker interaction with H-10, indicates that H-9 and H-8 are located on the same face of the six-membered ring. The absolute configuration of compound **5** was determined as 8R, 9*R*, 12*S*, 13*R*, 14*R*, 15*S*, 19*R* through comparative analysis of the experimental ECD spectrum (Fig. 4) with the calculated ECD spectrum. Therefore, compound **5** was named talarine O.

Compound **6**, a colorless oil, exhibited the molecular formula C_25_H_31_ClO_8_ according to the HRESIMS data ([M + H]^+^ *m/z* 495.1775, calcd for C_25_H_32_O_8_Cl, 495.1786). The ^1^H NMR data (Table 2) displayed resonances for one oxygen-bearing methine protons at *δ*_H_ 4.70 (1H, m, H-8), and five methyl protons (four singlets and one doublet). The ^13^C NMR data (Table 1) showed a total of 25 carbon signals, including an ester carbonyl carbon at *δ*_C_ 171.7 (C-7), six aromatic carbons at *δ*_C_ 158.7 (C-6), 156.9 (C-4), 139.3 (C-2), 111.8 (C-3), 108.5 (C-5), and 103.0 (C-1), two ketal carbons at *δ*_C_ 100.5 (C-19) and 99.9 (C-15), one oxygen-bearing methine at *δ*_C_ 76.8 (C-8), six methylenes at *δ*_C_ 32.9 (C-17), 32.1 (C-16), 31.6 (C-9), 28.4 (C-11), 25.4 (C-20), and 23.7 (C-21), four nonprotonated carbons including three oxygenated at *δ*_C_ 81.5 (C-13), 78.8 (C-12), and 74.6 (C-23), and five methyls at *δ*_C_ 24.8 (C-24), 24.5 (C-25), 23.9 (C-18), 20.9 (C-10), and 19.2 (C-22). These data were nearly identical with those of **5**, except for the absence of an acetyl group at C-9, as proved by the ^1^H−^1^H COSY correlations (Fig. 2) of H_3_-10/H-8/H_2_-9 along with the HMBC correlations from H_3_-10 to C-8 and C-9. Additionally, the NOESY correlations (Fig. 3) of H_3_-18/H-11*β* (*δ*_H_ 2.92), H_3_-22/H-11*β*, H_3_-18/H-16*β* (*δ*_H_ 1.97), and H_3_-22/H-16*β* indicated that **6** and **5** had similar relative configurations. The absolute configuration of **6** was determined by the experimental ECD spectrumin comparison to calculated spectra (Fig. 4), confirming the absolute configuration of (8*R*,12*S*,13*R*,14*R*,15*S*,19*R*)-**6**. Therefore, compound **6** was named talarine P.

Compound **7** was isolated as a colorless oil, whose molecular formula was established as C_25_H_31_ClO_8_ by HRESIMS ([M + H]^+^ *m/z* 495.1779, calcd for C_25_H_32_O_8_Cl, 495.1786). The ^1^H NMR spectral data (Table 2) of **7** showed two oxygen-bearing methine protons at *δ*_H_ 4.70 (1H, d, *J* = 2.0 Hz, H-9) and 4.62 (1H, qd, *J* = 6.7, 1.8 Hz, H-8), as well as five methyl signals including four singlets at *δ*_H_ 1.35 (3H, s, H_3_-18), 1.19 (3H, s, H_3_-25), 1.06 (3H, s, H_3_-24), 1.02 (3H, s, H_3_-22), and one doublet at *δ*_H_ 1.57 (3H, d, *J* = 6.6 Hz, H_3_-10). The ^13^C NMR data (Table 1) showed 25 carbons, containing one ester carbonyl carbon at *δ*_C_ 171.4 (C-7), six aromatic carbons, one ketal carbon at *δ*_C_ 100.9 (C-19), two oxygen-bearing methines at *δ*_C_ 79.4 (C-8) and 63.9 (C-9), five methylenes at *δ*_C_ 35.1 (C-17), 30.8 (C-20), 30.4 (C-16), 28.2 (C-11), and 26.6 (C-21), five nonprotonated carbons containing three oxygen-bearing at *δ*_C_ 82.3 (C-13), 79.4 (C-12), and 76.5 (C-15), and five methyls at *δ*_C_ 25.0 (C-24), 24.5 (C-18), 19.6 (C-22), 18.7 (C-25), and 16.5 (C-10). The comparative NMR analysis (Tables 1 and 2) between **7** and **6** demonstrated their structural congruence, with the notable exceptions of an oxygenated methine and nonprotonated carbon appearing in **7**, while the ketal carbon and methylene group present in **6** were absent. ^1^H−^1^H COSY correlations (Fig. 2) of H_3_-10/H-8/H-9 and HMBC correlations from H-9 to C-1, C-2, and C-3, indicated that C-9 methylene in **6** was replaced by the oxygenated methine in **7**. Furthermore, the HMBC correlations from H_3_-24 to C-15, C-19, C-23, C-25, and H_3_-25 to C-15, C-23 suggested the linkage of C-15 and C-23. Finally, the relative configuration of **7** was further identified through the NOESY correlations (Fig. 3) of H_3_-10/H-9, H_3_-18/H-11*β* (*δ*_H_ 3.13), H-11*β*/H_3_-22, H_3_-18/H-16*β* (*δ*_H_ 1.81), H_3_-22/H-16*β*, and H-9/H-11*α* (*δ*_H_ 3.06). The absolute configuration of **7** was determined by the experimental ECD spectrumin comparison to calculated spectra (Fig. 4), confirming the absolute configuration of (8*R*,9*S*,12*S*,13*R*,14*S*,15*R*,19*R*)-**7.** Therefore, compound **7** was named talarine Q.

In addition to the seven novel compounds described above, five known compounds were also isolated (Fig. 1), including talaromyolide C (**8**) [6], talaromyolide H (**9**) [7], talarine C (**10**) [16], talarine A (**11**) [16] and talaromyolide J (**12**) [7]. All of these compounds were identified by comparing their ^1^H and ^13^C NMR data with those reported in the literatures.

### PTP1B inhibitory activity evaluation

This study systematically investigated the antidiabetic potential of compounds (**1**–**12**) by evaluating their inhibitory activity against PTP1B (Table 3). The results revealed that compounds **2**, **3**, **5** and **12** exhibited significant inhibitory effects with IC_50_ values of 1.74, 8.54, 6.57, and 3.03 μM, respectively. Compared to the positive control sodium vanadate (Na_3_VO_4_, IC_50_ = 0.83 μM), compound **2** (IC_50_ = 1.74 μM) displayed superior inhibitory efficacy. Notably, compounds **1**–**4** and **12** (IC_50_ = 1.74–17.60 μM) feature a pentacyclic system, while other compounds form peroxide rings at the C-15 and C-19. The former group demonstrated higher inhibitory activity compared to the latter. This observation suggests that the presence of peroxide rings at the C-15 and C-19 diminishes the inhibitory effects on PTP1B (Table 3).

**Table 3:** Inhibitory activities for compounds against PTP1B

Compounds	IC_50_ (μM)	Compounds	IC_50_ (μM)
**1**	16.48 ± 0.60	**8**	12.69 ± 1.30
**2**	1.74 ± 3.81	**9**	35.12 ± 3.30
**3**	17.60 ± 0.98	**10**	11.13 ± 1.31
**4**	8.54 ± 1.52	**11**	18.42 ± 2.16
**5**	6.57 ± 1.47	**12**	3.03 ± 2.25
**6**	14.26 ± 0.86	Na_3_VO_4_	0.83 ± 2.57
**7**	15.60 ± 1.60		

### Kinetic analysis and molecular docking

To further elucidate the inhibitory mechanisms of **2** and **12**, which exhibit significant inhibitory activity against PTP1B (Table 3), kinetic analysis and molecular docking simulations were performed. 

In the absence of the inhibitor, PTP1B catalyzed *p*-NPP with a *Km* of 6.82 μM (Fig. 5a). However, addition of compound **2** to the reaction shifted the *Km* to the right without affecting the V_*max*_. Accordingly, the *Km* was 13.31 μM and 22.13 μM at 5 μM and 10 μM compound **2**, respectively. The Lineweaver–Burk or double-reciprocal plot further showed that **2** inhibits PTP1B competitively (Fig. 5b). These findings demonstrate that **2** was indeed a competitive PTP1B inhibitor. In the case of **12**, PTP1B catalyzed *p*-NPP with a *Km* of 9.56 μM in control group (Fig. 5c). The trend lines (Fig. 5b and d) of **2** and **12** exhibit similarity and intersected on the Y-axis. A gradual increase in *Km* values accompanied by an unchanged V_*max*_ indicated that compounds **2** and **12** were competitive inhibitors of PTP1B.

The binding interactions of **2** and **12** with PTP1B are depicted in Fig. 6. The molecular docking analysis of **2** (Fig. 6a) against PTP1B revealed a thermodynamically favorable binding profile (*Δ*G = − 8.0 kcal/mol), indicative of strong target engagement. Key stabilizing interactions included that **2** formed Van der Waals interactions with THR165/GLU97/THR138/LEU140/ASN162/THR164 /GLU101/HIS60. This indicated a π-alkyl interactions with TRP100, and established hydrogen bonds with ARG169/LYS103. In the case of **12** (Fig. 6b), the hydroxyl group and two carbonyl groups established hydrogen bonds with residues LEU204/ARG199, respectively. Additionally, A hydrophobic cluster comprising ARG199 (alkyl) and PHE280 (π-alkyl), and formed Van der Waals interactions with GLY202/LYS239/ASP236/ILE281/GLU200/ARG79.

## Conclusions

In this study, twelve polycyclic meroterpenoids, including seven previously undescribed compounds (talarines K–Q), were isolated from the desert-derived fungus *Talaromyces* sp. HMT-8. Comprehensive structural elucidation was conducted using NMR, HRESIMS, and ECD techniques. Bioactivity assays demonstrated that several of these compounds exhibit notable inhibitory effects against PTP1B, an important therapeutic target for type 2 diabetes and obesity. Notably, compounds **2** and **12** showed potent PTP1B inhibition with IC₅₀ values of 1.74 and 3.03 μM, respectively, and were identified as competitive inhibitors through kinetic and molecular docking studies. These findings not only expand the chemical diversity of fungal meroterpenoids, but also highlight their potential as promising lead compounds for the development of novel antidiabetic agents.

## Materials and methods

### General experimental procedures

ECD spectra were measured on Bio-Logic MOS-450 spectropolarimeter. IR spectra were recorded on a Thermo Nicolet iS 10 spectrometer. The NMR spectra were recorded on a Bruker AM-600 spectrometer with TMS as an internal standard. HRESIMS spectra were obtained on a Thermo U3000 spectrometer fitted with an ESI source. Semipreparative HPLC was carried out using an ODS column (YMC-pack ODS-A, 10 × 250 mm, 5 μm, 2.5 mL/min). All solvents used were of analytical and chromatographic grade. Silica gel (200–300 mesh; Qingdao Haiyang Chemical Co., Ltd., Qingdao, Shandong, China), C18 reversed-phase silica gel (YMC ODS-A gel, YMC Co., Ltd., Kyoto, Japan).

### Fungal material

The HMT-8 was isolated from the desert soil in Alashan region in the west of Inner Mongolia Autonomous Region of China. The strain was identified as *Talaromyces* sp. based on microscopic examination and by internal transcribed spacer (ITS1–4) sequencing. The ITS sequence has been deposited in GenBank (http://www.ncbi.nlm.nih.gov) with accession number No. PV436706. The purified strain was cultivated in a PDA medium plate (containing 200 g potatoes, boil 20 min, take filtrate; 20 g glucose; 20 g agar in 1 L water) at 28 ℃ for 7 days. Then, it was cut into small pieces and cultured in PDB medium plate (containing 200 g potatoes, boil 20 min, take filtrate; 20 g glucose; in 1 L water) for 5 days.

### Fermentation, Extraction and Isolation

The fermentation was carried out in 400 flasks (500 mL), each containing 100 g of rice and 80 mL H_2_O, autoclaving at 15 psi for 30 min. After cooling to room temperature, each flask was inoculated with 20 mL of the spore inoculum and incubated at room temperature for 30 days.

The fermented material was extracted with EtOAc for three times, then the EtOAc solutions were combined and evaporated under reduced pressure to get 210 g of crude extract. The extract was fractionated by a silica gel VLC column using different solvents of increasing polarity, from EtOAc-petroleum ether (PE) to yield eleven fractions (Fr.1–11). Fr.4 was eluted with EtOAc-PE to obtain ten subfractions (Fr.4.1–4.10). Fr 4.3 was purified by a semipreparative reversed-phase (RP) HPLC column using MeCN/H_2_O = 43:57 (3 mL/min) to afford **7** (3.6 mg), **8** (4.9 mg) and **9** (6.2 mg). Fr 4.6 also purified by semi-preparative RP-HPLC using MeCN/H_2_O = 70:30 (3 mL/min) to yield **10** (5.7 mg) and **11** (4.8 mg). Fr.6 was separated using a silica gel VLC column (EtOAc-PE) to yield nine subfractions (Fr.6.1–6.9). Fr.6.2 was chromatographed by C18 reversed-phase (RP-18) silica gel using MeOH/H_2_O to collect subfractions (Fr.6.2.1–6.2.12). Fr.6.2.8 was separated by semi-preparative RP-HPLC using MeCN/H_2_O = 64:36 (3 mL/min) to afford **1** (5.4 mg), **2** (6.2 mg), **3** (5.7 mg), **4** (3.8 mg) and **5** (4.0 mg). Fr.6.2.11 was purified by semi-preparative RP-HPLC column using MeCN/H_2_O = 60:40 (4 mL/min) to afford **6** (4.6 mg). Fr.10 was purified by RP-18 using MeOH/H_2_O to collect six subfractions (Fr.10.1–10.6). Fr.10.3 and Fr.10.4 were also purified by semi-preparative RP-HPLC column using MeCN/H_2_O = 20:80 (3 mL/min) to afford **12** (5.2 mg).

Talarine K (**1**), yellow amorphous powder; [*α*]_D_^25^ –102 (*c* 0.1, MeOH); UV (MeOH) *λ*_max_ (log *ε*): 223 (3.21) nm, 274 (4.01) nm, 309 (3.22) nm; ECD (MeOH) *λ*_max_ (∆*ε*): 211 (+ 9.80), 235 (–7.50), 265 (5.13) nm; IR (KBr) *ν*_max_ (cm^−1^): 2933, 1701, 1478, 1326, 1165; ^1^H and ^13^C NMR data, see Tables 1 and 2; HR-ESI–MS *m/z*: 403.1748 [M + H]^+^ (Calcd. for C_22_H_27_O_7_, 403.1757).

Talarine L (**2**), yellow amorphous powder; [*α*]_D_^25^ –93 (*c* 0.1, MeOH); UV (MeOH) *λ*_max_ (log *ε*): 222 (3.01) nm, 278 (4.76) nm, 314 (3.15) nm; ECD (MeOH) *λ*_max_ (∆*ε*): 229 (–1.62), 253 (+ 0.16), 267 (–0.14) nm; IR (KBr) *ν*_max_ (cm^−1^): 2951, 1743, 1633, 1365, 1201; ^1^H and ^13^C NMR data, see Tables 1 and 2; HR-ESI–MS *m/z*: 443.2061 [M + H]^+^ (Calcd. for C_25_H_31_O_7_, 443.2070).

Talarine M (**3**), yellow amorphous powder; [*α*]_D_^25^ –112 (*c* 0.1, MeOH); UV (MeOH) *λ*_max_ (log *ε*): 219 (3.33) nm, 277 (4.15) nm, 313 (3.66) nm; ECD (MeOH) *λ*_max_ (∆*ε*): 217 (+ 2.40), 239 (− 2.80), 275 (+ 1.94) nm; IR (KBr) *ν*_max_ (cm^−1^): 2941, 1745, 1620, 1255, 1113; ^1^H and ^13^C NMR data, see Tables 1 and 2; HR-ESI–MS *m/z*: 491.1837 [M − H]^+^ (Calcd. for C_26_H_32_ClO_7_, 491.1843).

Talarine N (**4**), yellow amorphous powder; [*α*]_D_^25^ – 87 (*c* 0.05, MeOH); UV (MeOH) *λ*_max_ (log *ε*): 220 (3.16) nm, 277 (4.11) nm, 315 (3.01) nm; ECD (MeOH) *λ*_max_ (∆*ε*): 212 (+ 9.20), 238 (– 3.81), 272 (+ 2.59) nm; IR (KBr) *ν*_max_ (cm^−1^): 2930, 1712, 1689, 1257, 1099; ^1^H and ^13^C NMR data, see Tables 1 and 2; HR-ESI–MS *m/z*: 517.2438 [M + H]^+^ (Calcd. for C_28_H_37_O_9_, 517.2435).

Talarine O (**5**), white powder; [*α*]_D_^25^ – 121 (*c* 0.1, MeOH); UV (MeOH) *λ*_max_ (log *ε*): 223 (3.78) nm, 274 (4.66) nm, 319 (3.24) nm; ECD (MeOH) *λ*_max_ (∆*ε*): 227 (+ 14.07), 245 (− 2.18) nm, 260 (+ 1.97); IR (KBr) *ν*_max_ (cm^−1^): 2914, 1764, 1636, 1358, 1150; ^1^H and ^13^C NMR data, see Tables 1 and 2; HR-ESI–MS *m/z*: 553.1841 [M + H]^+^ (Calcd. for C_27_H_34_O_10_Cl, 553.1829).

Talarine P (**6**), white powder; [*α*]_D_^25^ –79 (*c* 0.05, MeOH); UV (MeOH) *λ*_max_ (log *ε*): 222 (4.01) nm, 269 (4.31) nm, 311 (3.11) nm; ECD (MeOH) *λ*_max_ (∆*ε*): 209 (+ 5.07), 239 (− 4.29), 278 (+ 2.74) nm; IR (KBr) *ν*_max_ (cm^−1^): 2942, 1733, 1616, 1369, 1104; ^1^H and ^13^C NMR data, see Tables 1 and 2; HR-ESI–MS *m/z*: 495.1775 [M + H]^+^ (Calcd. for C_25_H_32_O_8_Cl, 495.1786).

Talarine Q (**7**), white powder; [*α*]_D_^25^ – 67(*c* 0.1, MeOH); UV (MeOH) *λ*_max_ (log *ε*): 211 (+ 13.42) nm, 230 (–22.63) nm, 272 (24.49) nm; ECD (MeOH) *λ*_max_ (∆*ε*): 221 (7.04), 239 (− 2.33) nm; IR (KBr) *ν*_max_ (cm^−1^): 2913, 1688, 1436, 1356, 1138, 1007; ^1^H and ^13^C NMR data, see Tables 1 and 2; HR-ESI–MS *m/z*: 495.1779 [M + H]^+^ (Calcd. for C_25_H_32_O_8_Cl, 495.1786).

### Quantum chemistry calculations

The theoretical calculations of compounds were performed using Gaussian 09 [17]. The possible conformations were initially obtained from the program Spartan’14 and then optimized at B3LYP/6-31G* level in the gas phase. Room-temperature equilibrium populations were calculated according to the Boltzmann distribution law. The ECD calculations were performed using Time Dependent Density Functional Theory (TDDFT) [18] at wB97xd/ 6-311G** level in methanol with PCM model. The ECD spectra of compounds were obtained by weighing the Boltzmann distribution rate of each geometric conformation, and the sigma/gamma value for processing the calculated ECD was 0.3 eV [19]. All calculated curves were shifted + 15 nm to better simulate experimental spectra. The NMR calculations were performed using GIAO method at MPW1PW91/6-311G**// B3LYP/6-31G* level in METHANOL with PCM model [20]. The shielding constants (including ^13^C and ^1^H) obtained were directly performed statistical analyses with experimental chemical shifts by using DP4 + probability [21].

### PTP1B inhibitory assay

The p-nitrophenyl phosphate (*p*-NPP) was used as a substrate to evaluate enzyme inhibition assays, following a previously established protocol. The final assay volume was 100 μL. The 75 μL reaction buffer containing 50 mM HEPES, 100 mM NaCl, 1 mM EDTA, and 1 mM dithiothreitol, 10 μL protein, and 5 μL sample were added to a 96-well plate and incubated at room temperature for 15 min. Subsequently, 10 μL of reaction buffer containing *p*-NPP was added to the reaction system. Sodium orthovanadate (Na₃VO₄) and dimethyl sulfoxide (DMSO) were used as the positive and negative controls, respectively. Enzymatic activity was determined by measuring absorbance at 405 nm. The half-maximal inhibitory concentration (IC₅₀) values were obtained from three independent experiments. The percentage of inhibition was calculated using the following equation:

$$\left[ {\left( {{\text{OD}}_{{{\text{control}}}} - {\text{ OD}}_{{{\text{blank}}}} } \right) \, - \, \left( {{\text{OD}}_{{{\text{compound}}}} - {\text{ OD}}_{{{\text{blank}}}} } \right)} \right]/\left( {{\text{OD}}_{{{\text{control}}}} - {\text{ OD}}_{{{\text{blank}}}} } \right) \, \times { 1}00\%$$

### Inhibition kinetic analysis

To determine the type of inhibition exerted by the most effective compound against PTP1B, enzyme kinetics analyses were performed using varying concentrations of the substrate (2, 1,0.5, 0.25, 0.125, 0.0625, 0.03125 mM *p*-NPP for PTP1B) in the presence of different concentrations of the inhibitor. The experimental procedure followed that described in the enzyme inhibition assay. The data were represented using Lineweaver–Burk double-reciprocal plots. The inhibition constants (Ki and Kis) were determined from the secondary plots of the slopes and intercepts derived from the Lineweaver–Burk plots.

### Molecular docking

Molecular docking studies were carried out using the AutoDock software suite. The 3D conformations of compounds were generated and energy-minimized using the MM2 force field in Chem3D Ultra 11.0. The crystal structure of PTP1B (PDB ID: 2NT7) was retrieved from the RCSB Protein Data Bank. Prior to docking. All water molecules and co-crystallized ligands were removed. The hydrogen atoms were added to the protein structures using AutoDock tools (v1.5.6). Docking using AutoDock Vina 1.1.2. Default docking parameters were employed throughout the process. Visualization and analysis of docking poses were conducted using PyMOL 2.3.0 (https://pymol.org).

### Statistical analysis

All experiments were repeated three times (n = 3). The results are expressed as mean ± SD. Statistical analysis was performed using GraphPad Prism 8.0. The two-tailed Student’s *t*-test was used to assess significance.

## Supplementary Information

Additional file 1.

## Data Availability

The datasets used or analyzed during the current study are available from the corresponding author on reasonable request.

## References

[1] Matsuda Y, Abe I. Biosynthesis of fungal meroterpenoids. Nat Prod Rep. 2016;33(1):26–53. 26497360 10.1039/c5np00090d

[2] Yamazaki H, Nakayama W, Takahashi O, Kirikoshi R, Izumikawa Y, Iwasaki K, Toraiwa K, Ukai K, Rotinsulu H, Wewengkang DS, Sumilat DA, Mangindaan RE, Namikoshi M. Verruculides A and B, two new protein tyrosine phosphatase 1B inhibitors from an Indonesian ascidian-derived *Penicillium verruculosum*. Bioorg Med Chem Lett. 2015;25(16):3087–90. 26115570 10.1016/j.bmcl.2015.06.026

[3] Zhou H, Li L, Wang W, Che Q, Li D, Gu Q, Zhu T. Chrodrimanins I and J from the Antarctic moss-derived fungus *Penicillium funiculosum* GWT2-24. J Nat Prod. 2015;78(6):1442–5. 26046820 10.1021/acs.jnatprod.5b00103

[4] Kong FD, Ma QY, Huang SZ, Wang P, Wang JF, Zhou LM, Yuan JZ, Dai HF, Zhao YX. Chrodrimanins K-N and related meroterpenoids from the fungus *Penicillium* sp. SCS-KFD09 isolated from a marine worm, *Sipunculus nudus*. J Nat Prod. 2017;80(4):1039–47. 28212032 10.1021/acs.jnatprod.6b01061

[5] Bai T, Quan Z, Zhai R, Awakawa T, Matsuda Y, Abe I. Elucidation and heterologous reconstitution of chrodrimanin B biosynthesis. Org Lett. 2018;20(23):7504–8. 30417647 10.1021/acs.orglett.8b03268

[6] Cao X, Shi Y, Wu X, Wang K, Huang S, Sun H, Dickschat JS, Wu B. Talaromyolides A-D and talaromytin: polycyclic meroterpenoids from the fungus *Talaromyces* sp. CX11. Org Lett. 2019;21(16):6539–42. 31364857 10.1021/acs.orglett.9b02466

[7] Cao X, Shi YT, Wu SH, Wang KW, Sun HX, He S, Diskschat JS, Wu B. Polycyclic meroterpenoids, talaromyolides E-K for antiviral activity against pseudorabies virus from the endophytic fungus *Talaromyces purpureogenus*. Tetrahedron. 2020;76(30):131349–57.

[8] Ding HE, Yang ZD, Sheng L, Zhou SY, Li S, Yao XJ, Zhi KK, Wang YG, Zhang F. Secovironolide, a novel furanosteroid scaffold with a five-membered B ring from the endophytic fungus *Talaromyces wortmannii* LGT-4. Tetrahedron Lett. 2015;56(48):6754–7.

[9] Kaur A, Raja HA, Swenson DC, Agarwal R, Deep G, Falkinham JO 3rd, Oberlies NH. Talarolutins A-D: meroterpenoids from an endophytic fungal isolate of *Talaromyces minioluteus*. Phytochemistry. 2016;126:4–10. 27048854 10.1016/j.phytochem.2016.03.013 PMC4861051

[10] Clark TA, Heyliger CE, Edel AL, Goel DP, Pierce GN. Codelivery of a tea extract prevents morbidity and mortality associated with oral vanadate therapy in streptozotocin-induced diabetic rats. Metabolism. 2004;53(9):1145–51. 15334376 10.1016/j.metabol.2004.03.017

[11] Singh S, Singh Grewal A, Grover R, Sharma N, Chopra B, Kumar Dhingra A, Arora S, Redhu S, Lather V. Recent updates on development of protein-tyrosine phosphatase 1B inhibitors for treatment of diabetes, obesity and related disorders. Bioorg Chem. 2022;121: 105626. 35255350 10.1016/j.bioorg.2022.105626

[12] Elchebly M, Payette P, Michaliszyn E, Cromlish W, Collins S, Loy AL, Normandin D, Cheng A, Himms-Hagen J, Chan CC, Ramachandran C, Gresser MJ, Tremblay ML, Kennedy BP. Increased insulin sensitivity and obesity resistance in mice lacking the protein tyrosine phosphatase-1B gene. Science. 1999;283(5407):1544–8. 10066179 10.1126/science.283.5407.1544

[13] Zinker BA, Rondinone CM, Trevillyan JM, Gum RJ, Clampit JE, Waring JF, Xie N, Wilcox D, Jacobson P, Frost L, Kroeger PE, Reilly RM, Koterski S, Opgenorth TJ, Ulrich RG, Crosby S, Butler M, Murray SF, McKay RA, Bhanot S, Monia BP, Jirousek MR. PTP1B antisense oligonucleotide lowers PTP1B protein, normalizes blood glucose, and improves insulin sensitivity in diabetic mice. Proc Natl Acad Sci USA. 2002;99(17):11357–62. 12169659 10.1073/pnas.142298199 PMC123261

[14] Fischer G, Müller T, Ostrowski R, Dott W. Mycotoxins of *Aspergillus fumigatus* in pure culture and in native bioaerosols from compost facilities. Chemosphere. 1999;38(8):1745–55. 10101846 10.1016/s0045-6535(98)00391-9

[15] Li X, Awakawa T, Mori T, Ling M, Hu D, Wu B, Abe I. Heterodimeric non-heme iron enzymes in fungal meroterpenoid biosynthesis. J Am Chem Soc. 2021;143(50):21425–32. 34881885 10.1021/jacs.1c11548

[16] Ren M, Li Z, Wang Z, Han W, Wang F, Li Y, Zhang W, Liu X, Zhang J, Luo DQ. Antiviral chlorinated drimane meroterpenoids from the fungus *Talaromyces pinophilus* LD-7 and their biosynthetic pathway. J Nat Prod. 2024;87(8):2034–44. 39126395 10.1021/acs.jnatprod.4c00539

[17] Frisch MJ, Trucks GW, Schlegel HB, Scuseria GE, Robb MA, Cheeseman JR, Scalmani G, Barone V, Mennucci B, Petersson GA, Nakatsuji H, Caricato M, Li X, Hratchian HP, Izmaylov, AF, Bloino J, Zheng G, Sonnenberg JL, Hada M, Ehara M, Toyota K, Fukuda R, Hasegawa J, Ishida M, Nakajima T, Honda Y, Kitao O, Nakai H, Vreven T, Montgomery JA, Peralta JE, Ogliaro F, Bearpark M, Heyd JJ, Brothers E, Kudin KN, Staroverov VN, Keith T, Kobayashi R, Normand J, Raghavachari K, Rendell A, Burant JC, Iyengar SS, Tomasi J, Cossi M, Rega N, Millam JM, Klene M, Knox JE, Cross JB, Bakken V, Adamo C, Jaramillo J, Gomperts R, Stratmann RE, Yazyev O, Austin AJ, Cammi R, Pomelli C, Ochterski JW, Martin RL, Morokuma K, Zakrzewski VG, Voth GA, Salvador P, Dannenberg JJ, Dapprich S, Daniels AD, Farkas O, Foresman JB, Ortiz JV, Cioslowski J, Fox DJ. 2013. Gaussian 09, Revision E.01. Gaussian, Inc., Wallingford CT.

[18] Bruhn T, Schaumlöffel A, Hemberger Y. SpecDis, Version 1.64. Wuerzburg: University of Wuerzburg; 2015.

[19] Srebro-Hooper M, Autschbach J. Calculating natural optical activity of molecules from first principles. Annu Rev Phys Chem. 2017;68:399–420. 28463650 10.1146/annurev-physchem-052516-044827

[20] Lodewyk MW, Siebert MR, Tantillo DJ. Computational prediction of ^1^H and ^13^C chemical shifts: a useful tool for natural product, mechanistic, and synthetic organic chemistry. Chem Rev. 2012;112(3):1839–62. 22091891 10.1021/cr200106v

[21] Zanardi MM, Sarotti AM. Sensitivity analysis of DP4+ with the probability distribution terms: development of a universal and customizable method. J Org Chem. 2021;86(12):8544–8. 34101443 10.1021/acs.joc.1c00987

