# Supplementary material for: Novel polycyclic meroterpenoids with protein tyrosine phosphatase 1B inhibitory activity isolated from desert-derived fungi Talaromyces sp. HMT-8
Source: Nat Prod Bioprospect. 2025 Jul 18;15(1):46. doi: 10.1007/s13659-025-00530-x (PMC12274187; doi:10.1007/s13659-025-00530-x)
Supplement: Supplementary file 1 — Additional file 1. [file 13659_2025_530_MOESM1_ESM.doc]

**Supporting Information**

**Novel Polycyclic meroterpenoids with Protein Tyrosine Phosphatase 1B Inhibitory Activity Isolated from Desert-Derived Fungi *Talaromyces* sp. HMT-8**

**Xin-yi Zhai ^a^, Jin-jie Liu ^b^, Cui-duan Wang ^a^, Yi-fan Dou ^a^, Jian-hua Lv ^a^, Li-an Wang ^a^, Jin-xiu Zhang ^a*^, Zhuang Li ^a*^**

^a^ *College of Life Sciences, Hebei Normal University, Shijiazhuang 050000, P. R. China*

^b^ *School of Land Science and Space Planning, Hebei GEO University,, Shijiazhuang 050000, P. R. China*

^*^correspondence author

**Figure S1**. ^1^H-NMR (600 MHz, CD_3_OD) spectrum of compound **1**

**Figure S2**. ^13^C-NMR (150 MHz, CD_3_OD) spectrum of compound **1**

**Figure S3**. HSQC (600 MHz, CD_3_OD) spectrum of compound **1**

**Figure S4**. HMBC (600 MHz, CD_3_OD) spectrum of compound **1**

**Figure S5**. ^1^H-^1^H COSY (600 MHz, CD_3_OD) spectrum of compound **1**

**Figure S6**. NOESY (600 MHz, CD_3_OD) spectrum of compound **1**

**Figure S7**. HRESIMS spectrum of **1**

**Figure S8**. ^1^H-NMR (600 MHz, CD_3_OD) spectrum of compound **2**

**Figure S9**. ^13^C-NMR (150 MHz, CD_3_OD) spectrum of compound **2**

**Figure S10**. HSQC (600 MHz, CD_3_OD) spectrum of compound **2**

**Figure S11**. HMBC (600 MHz, CD_3_OD) spectrum of compound **2**

**Figure S12**. ^1^H-^1^H COSY (600 MHz, CD_3_OD) spectrum of compound **2**

**Figure S13**. NOESY (600 MHz, CD_3_OD) spectrum of compound **2**

**Figure S14**. HRESIMS spectrum of **2**

**Figure S15**. ^1^H-NMR (600 MHz, CD_3_OD) spectrum of compound **3**

**Figure S16**. ^13^C-NMR (150 MHz, CD_3_OD) spectrum of compound **3**

**Figure S17**. HSQC (600 MHz, CD_3_OD) spectrum of compound **3**

**Figure S18**. HMBC (600 MHz, CD_3_OD) spectrum of compound **3**

**Figure S19**. ^1^H-^1^H COSY (600 MHz, CD_3_OD) spectrum of compound **3**

**Figure S20**. NOESY (600 MHz, CD_3_OD) spectrum of compound **3**

**Figure S21**. HRESIMS spectrum of **3**

**Figure S22**. ^1^H-NMR (600 MHz, CD_3_OD) spectrum of compound **4**

**Figure S23**. ^13^C-NMR (150 MHz, CD_3_OD) spectrum of compound **4**

**Figure S24**. HSQC (600 MHz, CD_3_OD) spectrum of compound **4**

**Figure S25**. HMBC (600 MHz, CD_3_OD) spectrum of compound **4**

**Figure S26**. ^1^H-^1^H COSY (600 MHz, CD_3_OD) spectrum of compound **4**

**Figure S27**. NOESY (600 MHz, CD_3_OD) spectrum of compound **4**

**Figure S28**. HRESIMS spectrum of **4**

**Figure S29**. ^1^H-NMR (600 MHz, CD_3_OD) spectrum of compound **5**

**Figure S30**. ^13^C-NMR (150 MHz, CD_3_OD) spectrum of compound **5**

**Figure S31**. HSQC (600 MHz, CD_3_OD) spectrum of compound **5**

**Figure S32**. HMBC (600 MHz, CD_3_OD) spectrum of compound **5**

**Figure S33**. ^1^H-^1^H COSY (600 MHz, CD_3_OD) spectrum of compound **5**

**Figure S34**. NOESY (600 MHz, CD_3_OD) spectrum of compound **5**

**Figure S35**. HRESIMS spectrum of **5**

**Figure S36**. ^1^H-NMR (600 MHz, CD_3_OD) spectrum of compound **6**

**Figure S37**. ^13^C-NMR (150 MHz, CD_3_OD) spectrum of compound **6**

**Figure S38**. HSQC (600 MHz, CD_3_OD) spectrum of compound **6**

**Figure S39**. HMBC (600 MHz, CD_3_OD) spectrum of compound **6**

**Figure S40**. ^1^H-^1^H COSY (600 MHz, CD_3_OD) spectrum of compound **6**

**Figure S41**. NOESY (600 MHz, CD_3_OD) spectrum of compound **6**

**Figure S42**. HRESIMS spectrum of **6**

**Figure S43**. ^1^H-NMR (600 MHz, CD_3_OD) spectrum of compound **7**

**Figure S44**. ^13^C-NMR (150 MHz, CD_3_OD) spectrum of compound **7**

**Figure S45**. HSQC (600 MHz, CD_3_OD) spectrum of compound **7**

**Figure S46**. HMBC (600 MHz, CD_3_OD) spectrum of compound **7**

**Figure S47**. ^1^H-^1^H COSY (600 MHz, CD_3_OD) spectrum of compound **7**

**Figure S48**. NOESY (600 MHz, CD_3_OD) spectrum of compound **7**

**Figure S49**. HRESIMS spectrum of **7**

**Figure S50.** Detailed DP4+ probability of **3** calculated at mPW1PW91/6-311G** level in METHANOL with PCM model.

**Table S1** Important thermodynamic parameters and Boltzmann distributions of the optimized **2** at b3lyp/6-31g* level in gas phase.

**Table S2**. The coordinates for the lowest energy conformers of **2**.

**Table S3**. Important thermodynamic parameters and Boltzmann distributions of the optimized **3** at b3lyp/6-31g* level in gas phase.

**Table S4**. The coordinates for the lowest energy conformers of **3**.

**Table S5**. Important thermodynamic parameters and Boltzmann distributions of the optimized **4** at b3lyp/6-31g* level in gas phase.

**Table S6**. The coordinates for the lowest energy conformers of **4**.

**Table S7**. Important thermodynamic parameters and Boltzmann distributions of the optimized **5** at b3lyp/6-31g* level in gas phase.

**Table S8**. The coordinates for the lowest energy conformers of **5**.

**Table S9**. Important thermodynamic parameters and Boltzmann distributions of the optimized **6** at b3lyp/6-31g* level in gas phase.

**Table S10**. The coordinates for the lowest energy conformers of **6**.

**Table S11**. Important thermodynamic parameters and Boltzmann distributions of the optimized **7** at b3lyp/6-31g* level in gas phase.

**Table S12**. The coordinates for the lowest energy conformers of **7**.

**Figure S1**. ^1^H-NMR (600 MHz, CD_3_OD) spectrum of compound **1**

**Figure S2**. ^13^C-NMR (150 MHz, CD_3_OD) spectrum of compound **1**

**Figure S3**. HSQC (600 MHz, CD_3_OD) spectrum of compound **1**

**Figure S4**. HMBC (600 MHz, CD_3_OD) spectrum of compound **1**

**Figure S5**. ^1^H-^1^H COSY (600 MHz, CD_3_OD) spectrum of compound **1**

**Figure S6**. NOESY (600 MHz, CD_3_OD) spectrum of compound **1**

**Figure S7**. HRESIMS spectrum of **1**

**Figure S8**. ^1^H-NMR (600 MHz, CD_3_OD) spectrum of compound **2**

**Figure S9**. ^13^C-NMR (150 MHz, CD_3_OD) spectrum of compound **2**

**Figure S10**. HSQC (600 MHz, CD_3_OD) spectrum of compound **2**

**Figure S11**. HMBC (600 MHz, CD_3_OD) spectrum of compound **2**

**Figure S12**. ^1^H-^1^H COSY (600 MHz, CD_3_OD) spectrum of compound **2**

**Figure S13**. NOESY (600 MHz, CD_3_OD) spectrum of compound **2**

**Figure S14**. HRESIMS spectrum of **2**

**Figure S15**. ^1^H-NMR (600 MHz, CD_3_OD) spectrum of compound **3**

**Figure S16**. ^13^C-NMR (150 MHz, CD_3_OD) spectrum of compound **3**

**Figure S17**. HSQC (600 MHz, CD_3_OD) spectrum of compound **3**

**Figure S18**. HMBC (600 MHz, CD_3_OD) spectrum of compound **3**

**Figure S19**. ^1^H-^1^H COSY (600 MHz, CD_3_OD) spectrum of compound **3**

**Figure S20**. NOESY (600 MHz, CD_3_OD) spectrum of compound **3**

**Figure S21**. HRESIMS spectrum of **3**

**Figure S22**. ^1^H-NMR (600 MHz, CD_3_OD) spectrum of compound **4**

**Figure S23**. ^13^C-NMR (150 MHz, CD_3_OD) spectrum of compound **4**

**Figure S24**. HSQC (600 MHz, CD_3_OD) spectrum of compound **4**

**Figure S25**. HMBC (600 MHz, CD_3_OD) spectrum of compound **4**

**Figure S26**. ^1^H-^1^H COSY (600 MHz, CD_3_OD) spectrum of compound **4**

**Figure S27**. NOESY (600 MHz, CD_3_OD) spectrum of compound **4**

**Figure S28**. HRESIMS spectrum of **4**

**Figure S29**. ^1^H-NMR (600 MHz, CD_3_OD) spectrum of compound **5**

**Figure S30**. ^13^C-NMR (150 MHz, CD_3_OD) spectrum of compound **5**

**Figure S31**. HSQC (600 MHz, CD_3_OD) spectrum of compound **5**

**Figure S32**. HMBC (600 MHz, CD_3_OD) spectrum of compound **5**

**Figure S33**. ^1^H-^1^H COSY (600 MHz, CD_3_OD) spectrum of compound **5**

**Figure S34**. NOESY (600 MHz, CD_3_OD) spectrum of compound **5**

**Figure S35**. HRESIMS spectrum of **5**

**Figure S36**. ^1^H-NMR (600 MHz, CD_3_OD) spectrum of compound **6**

**Figure S37**. ^13^C-NMR (150 MHz, CD_3_OD) spectrum of compound **6**

**Figure S38**. HSQC (600 MHz, CD_3_OD) spectrum of compound **6**

**Figure S39**. HMBC (600 MHz, CD_3_OD) spectrum of compound **6**

**Figure S40**. ^1^H-^1^H COSY (600 MHz, CD_3_OD) spectrum of compound **6**

**Figure S41**. NOESY (600 MHz, CD_3_OD) spectrum of compound **6**

**Figure S42**. HRESIMS spectrum of **6**

**Figure S43**. ^1^H-NMR (600 MHz, CD_3_OD) spectrum of compound **7**

**Figure S44**. ^13^C-NMR (150 MHz, CD_3_OD) spectrum of compound **7**

**Figure S45**. HSQC (600 MHz, CD_3_OD) spectrum of compound **7**

**Figure S46**. HMBC (600 MHz, CD_3_OD) spectrum of compound **7**

**Figure S47**. ^1^H-^1^H COSY (600 MHz, CD_3_OD) spectrum of compound **7**

**Figure S48**. NOESY (600 MHz, CD_3_OD) spectrum of compound **7**

**Figure S49**. HRESIMS spectrum of **7**

**Figure S50.** Detailed DP4+ probability of **3** calculated at mPW1PW91/6-311G** level in METHANOL with PCM model.

**Table S1** Important thermodynamic parameters and Boltzmann distributions of the optimized **2** at b3lyp/6-31g* level in gas phase.

| Conformations | Energy (a.u) | Δ G(kcal/mol) | % |
| --- | --- | --- | --- |
| 1 | -1496.998407 | 0 | 70.49% |
| 2 | -1496.997048 | 0.85 | 16.71% |
| 3 | -1496.996563 | 1.16 | 10.00% |
| 4 | -1496.994843 | 2.24 | 1.62% |
| 5 | -1496.994552 | 2.42 | 1.19% |

**Table S2**. The coordinates for the lowest energy conformers of **2**.

Energy = -1496.99840700 a.u. #Cluster: 1

C	1.56409200	1.65429400	0.62654300

O	-4.77547700	0.69965900	-0.93408900

C	-3.65196000	1.53883100	-1.29913800

C	-2.47775700	0.68323800	-1.75245200

C	-2.11674000	-0.32100700	-0.68512000

C	-3.17870100	-0.78688300	0.11997300

C	-4.55356700	-0.32863100	-0.08824000

C	-0.81348400	-0.77729800	-0.49884000

C	-0.58411000	-1.70722000	0.54153200

C	-1.61570100	-2.18585700	1.34349700

C	-2.92252500	-1.75140100	1.13230400

O	-5.52947900	-0.84096500	0.45912000

C	-4.16164100	2.49553400	-2.36043000

O	-3.89489300	-2.26333900	1.89867700

C	0.35095200	-0.26315400	-1.30955200

C	1.66248700	-0.45558800	-0.54563200

C	1.76629000	-1.91869700	-0.03639000

O	0.65070200	-2.17595500	0.85118300

C	2.91643500	0.08420400	-1.30120400

C	4.14435100	-0.16627500	-0.39582100

C	4.28180800	-1.56935500	0.18095700

C	2.99988800	-2.07011600	0.86065300

C	1.70408000	-3.00055900	-1.11857900

O	1.56543800	0.28252300	0.69737600

C	2.08581900	2.31600000	-0.40800400

C	2.73824600	1.59898200	-1.55013800

C	3.21425500	-0.56942100	-2.66916300

C	0.90646500	2.27540700	1.84798600

C	-0.61557600	2.11621500	1.76316200

C	1.45100400	1.63106600	3.13213700

O	1.14712500	3.68389600	1.85988800

O	4.98443500	0.68821600	-0.20027700

H	-3.35790900	2.09434200	-0.39803100

H	-2.74558000	0.16831400	-2.68673600

H	-1.63656300	1.34235800	-1.98556800

H	-1.40424800	-2.91012800	2.12073600

H	-3.37631500	3.20824300	-2.63313500

H	-5.02539000	3.05282700	-1.98752100

H	-4.46513000	1.94620400	-3.25768700

H	-4.74982800	-1.88969100	1.55818000

H	0.41098700	-0.75662100	-2.28671200

H	0.23083700	0.80326700	-1.51021200

H	4.55920800	-2.24274900	-0.64179300

H	5.12006100	-1.56040400	0.88174000

H	2.81778100	-1.49320800	1.76933700

H	3.10741800	-3.12010500	1.15231200

H	0.85178400	-2.86078500	-1.78819900

H	2.61105800	-3.04970400	-1.72150800

H	1.57927300	-3.96302600	-0.61474700

H	2.02646900	3.39750400	-0.41943100

H	2.16216900	1.74476100	-2.47575800

H	3.73302300	2.01764600	-1.73534100

H	2.32556900	-0.57414300	-3.30675400

H	3.98413700	0.02189600	-3.17525700

H	3.58396600	-1.59269200	-2.59945700

H	-1.08277500	2.56806500	2.64366600

H	-0.99072800	2.62487600	0.86940500

H	-0.89137500	1.06055000	1.71312500

H	2.53952800	1.75347500	3.19241400

H	1.22902800	0.56145400	3.16068700

H	0.99549100	2.11675800	4.00023000

H	2.10877800	3.80458300	1.92675400

Energy = -1496.99704800 a.u. #Cluster: 2

C	-1.60203900	1.81403300	0.07115100

O	4.69971000	0.55937500	1.25072000

C	3.77940000	0.15429000	2.29610800

C	2.33223000	0.35378200	1.85708400

C	2.06740000	-0.30468800	0.52714400

C	3.14292500	-0.34008200	-0.38247500

C	4.46640400	0.17311900	-0.01969500

C	0.80041400	-0.76399300	0.17417000

C	0.61500400	-1.23115300	-1.14660100

C	1.65754500	-1.26153900	-2.06979500

C	2.92743000	-0.81675000	-1.70484500

O	5.38804300	0.28780600	-0.82760600

C	4.09785900	-1.27587600	2.72182600

O	3.89946600	-0.84590100	-2.62501500

C	-0.38132400	-0.66480000	1.11006000

C	-1.68064400	-0.59230600	0.30502400

C	-1.72410700	-1.74994500	-0.73124000

O	-0.59205300	-1.62098100	-1.62639600

C	-2.96610000	-0.42987400	1.17622500

C	-4.18014000	-0.36704300	0.21960300

C	-4.24961300	-1.44096100	-0.85778300

C	-2.94135400	-1.58114800	-1.64658400

C	-1.63149100	-3.16179700	-0.14468900

O	-1.59648200	0.57729300	-0.54496000

C	-2.19634600	2.00041900	1.24951200

C	-2.86659700	0.88146700	1.98669900

C	-3.24219100	-1.56597000	2.18707000

C	-0.79056900	2.80295600	-0.74634400

C	-1.29856000	2.84650600	-2.19532500

C	-0.77752100	4.19790700	-0.13149600

O	0.58056900	2.36406400	-0.72416100

O	-5.06277400	0.45513000	0.35630300

H	4.01121000	0.83989300	3.11496700

H	1.67731500	-0.03642100	2.64133600

H	2.11136000	1.42522400	1.75356300

H	1.47560100	-1.61024600	-3.07905400

H	5.16017500	-1.36819400	2.96387900

H	3.85922600	-1.98613600	1.92392900

H	3.51324900	-1.54425900	3.60870800

H	4.69635000	-0.42439700	-2.20565800

H	-0.41623800	-1.50891000	1.80883700

H	-0.30398600	0.24143800	1.71297400

H	-4.49798200	-2.39235300	-0.36735600

H	-5.08394100	-1.19539700	-1.51900700

H	-2.77862200	-0.68386200	-2.24681900

H	-2.99649000	-2.43268200	-2.33274600

H	-0.78663900	-3.26295200	0.54119000

H	-2.53810200	-3.46614000	0.37810700

H	-1.47167300	-3.85332300	-0.97651100

H	-2.17784300	2.97981200	1.71177400

H	-2.33078900	0.68203300	2.92647000

H	-3.88577800	1.16867800	2.26519300

H	-2.35207700	-1.78892700	2.78235600

H	-4.02856900	-1.23562500	2.87325700

H	-3.58233000	-2.49434300	1.72745400

H	-1.27189800	1.84853700	-2.64251600

H	-2.33003500	3.21317300	-2.23972300

H	-0.65838400	3.51079400	-2.78344000

H	-1.78630800	4.61791100	-0.08348500

H	-0.15324900	4.85119200	-0.74721300

H	-0.35062800	4.16991900	0.87492300

H	0.59715300	1.46194500	-1.08528000

Energy = -1496.99656300 a.u. #Cluster: 3

C	1.78510200	1.68609600	-0.46721800

O	-4.79459000	0.29684900	-1.38895200

C	-3.89800300	-0.44092700	-2.25836100

C	-2.44264300	-0.11246300	-1.93454000

C	-2.15727800	-0.32049100	-0.46765100

C	-3.22122100	-0.06951300	0.42265500

C	-4.54336500	0.32905200	-0.06278100

C	-0.89594000	-0.68489200	-0.00697100

C	-0.71225100	-0.80340200	1.38908000

C	-1.74088800	-0.54300500	2.29000700

C	-2.99705600	-0.15843500	1.82379300

O	-5.44805800	0.71167600	0.67885700

C	-4.22282600	-1.92959500	-2.18074800

O	-3.95494800	0.10871900	2.72149200

C	0.27868700	-0.85141500	-0.94107800

C	1.59577000	-0.70495000	-0.17537900

C	1.56371800	-1.58999800	1.10086600

O	0.47883900	-1.13489600	1.94666700

C	2.86380400	-0.86044100	-1.07247700

C	4.09930100	-0.67818100	-0.16135500

C	4.11096600	-1.49394600	1.12396900

C	2.82078600	-1.33787900	1.94106900

C	1.31807500	-3.08221000	0.86075000

O	1.63652900	0.62560700	0.39346600

C	2.32777500	1.55120600	-1.67842700

C	2.84106000	0.23106100	-2.16617900

C	3.02071100	-2.21988800	-1.79048700

C	1.25652200	2.97318100	0.14381800

C	-0.27594400	2.95752900	0.14767800

C	1.80554700	3.15098900	1.56776400

O	1.63164100	4.08393500	-0.67281900

O	5.03290300	0.03128300	-0.47601100

H	-4.14575200	-0.06405700	-3.25376400

H	-1.79642000	-0.73292000	-2.56241200

H	-2.23710300	0.93375500	-2.20183300

H	-1.55964900	-0.62139200	3.35510000

H	-5.28940700	-2.09045300	-2.35983900

H	-3.96851000	-2.33712100	-1.19754900

H	-3.65480700	-2.47887700	-2.93965200

H	-4.74587200	0.41811800	2.20606500

H	0.25266500	-1.81988900	-1.45520900

H	0.24799500	-0.08466500	-1.71835000

H	4.25925400	-2.54819800	0.85293700

H	4.98343600	-1.18742600	1.70592700

H	2.75673300	-0.32004100	2.33061000

H	2.82566600	-2.01970100	2.79801500

H	0.43492900	-3.25393700	0.24027400

H	2.16844500	-3.58156200	0.39621700

H	1.13932000	-3.54900400	1.83332800

H	2.38802000	2.41922100	-2.32354500

H	2.24040100	-0.12492200	-3.01619700

H	3.86578200	0.34183000	-2.53639500

H	2.10287200	-2.49692900	-2.31677900

H	3.81710700	-2.12814500	-2.53606600

H	3.29083500	-3.04245800	-1.12771800

H	-0.65392000	3.88387200	0.59097700

H	-0.64748900	2.88565300	-0.87922000

H	-0.65097000	2.10713500	0.72173100

H	2.90250100	3.16717800	1.55780000

H	1.48531800	2.33383500	2.21893800

H	1.44521400	4.09944500	1.97713900

H	2.60243100	4.12137500	-0.66893100

Energy = -1496.99484300 a.u. #Cluster: 4

C	1.85185500	1.70103300	-0.48355400

O	-4.80578200	0.27019400	-1.41304300

C	-3.90129000	-0.47772800	-2.26530100

C	-2.44941300	-0.13347800	-1.94217500

C	-2.16827600	-0.31060900	-0.47056500

C	-3.23731900	-0.05282600	0.41124800

C	-4.56098100	0.32669900	-0.08651100

C	-0.90441000	-0.65305700	0.00097900

C	-0.72553900	-0.74437900	1.39949100

C	-1.75934600	-0.47755500	2.29208200

C	-3.01754600	-0.11299400	1.81458600

O	-5.47116800	0.71526400	0.64474100

C	-4.21707600	-1.96675100	-2.16168000

O	-3.98045100	0.16184400	2.70385500

C	0.27310900	-0.82693700	-0.92867800

C	1.59208800	-0.69411700	-0.16380400

C	1.54236300	-1.55233800	1.13046900

O	0.46957900	-1.05043000	1.96593800

C	2.85667100	-0.89237600	-1.05675700

C	4.10735500	-0.75122100	-0.15724000

C	4.08930000	-1.52932700	1.15127100

C	2.80611900	-1.31237300	1.96332900

C	1.26115500	-3.04260600	0.92056500

O	1.65447500	0.64517500	0.37894500

C	2.40944400	1.53362000	-1.68257200

C	2.87119600	0.18940000	-2.15922400

C	2.97175300	-2.26281400	-1.76302300

C	1.30837800	2.97353100	0.14816000

C	1.91365000	4.22987400	-0.46916300

C	-0.22793200	2.98143900	0.03426900

O	1.68255600	2.99710600	1.53030800

O	5.07986200	-0.11566200	-0.50766100

H	-4.14766400	-0.12094700	-3.26842400

H	-1.79622500	-0.76161100	-2.55512200

H	-2.24976600	0.90878500	-2.22902400

H	-1.58160200	-0.53545800	3.35910100

H	-5.28161700	-2.13750200	-2.34360900

H	-3.96611600	-2.35467500	-1.16972100

H	-3.64159200	-2.52649600	-2.90716900

H	-4.77265600	0.45497500	2.18114000

H	0.23693800	-1.79303900	-1.44626600

H	0.25233400	-0.05706300	-1.70411100

H	4.20436100	-2.59439200	0.90703800

H	4.97178000	-1.23646600	1.72490500

H	2.77538400	-0.28236600	2.32450200

H	2.79103800	-1.96906400	2.83951300

H	0.37811900	-3.20652200	0.29784900

H	2.10210200	-3.57217100	0.47255500

H	1.06568400	-3.48417300	1.90167500

H	2.54896500	2.38959900	-2.33198500

H	2.25743100	-0.15117900	-3.00629400

H	3.89913000	0.26007700	-2.52932400

H	2.05230300	-2.51018700	-2.30098600

H	3.78209100	-2.20586700	-2.49671200

H	3.20164200	-3.08915500	-1.08971900

H	1.55651000	5.10282200	0.08448000

H	3.00460900	4.19853700	-0.40650100

H	1.61767300	4.34120200	-1.51659100

H	-0.65314400	2.09185200	0.50834000

H	-0.54638100	2.99729000	-1.01457300

H	-0.62474300	3.86743900	0.53932000

H	1.41899700	2.13518000	1.89384700

Energy = -1496.99455200 a.u. #Cluster: 5

C	1.78136300	1.68746900	-0.48866000

O	-4.79214500	0.28618700	-1.39403600

C	-3.89666200	-0.46024800	-2.25739000

C	-2.44085600	-0.13212900	-1.93543600

C	-2.15651500	-0.32894600	-0.46668300

C	-3.22138400	-0.07201000	0.42119600

C	-4.54208700	0.32582100	-0.06768000

C	-0.89527000	-0.68904400	-0.00322200

C	-0.71238800	-0.79834000	1.39375500

C	-1.74268300	-0.53446900	2.29234200

C	-2.99830200	-0.15247400	1.82292700

O	-5.44673300	0.71577400	0.67049800

C	-4.22452600	-1.94761200	-2.16847800

O	-3.95704000	0.12087800	2.71830700

C	0.28068500	-0.85720500	-0.93523700

C	1.59710200	-0.69950000	-0.16996300

C	1.56753600	-1.57565600	1.11242600

O	0.47861100	-1.12155600	1.95448700

C	2.86504100	-0.86245400	-1.06642300

C	4.10504900	-0.67994000	-0.16120200

C	4.11297900	-1.47451200	1.13810500

C	2.82213100	-1.31102500	1.95165900

C	1.32929800	-3.07078800	0.88327200

O	1.63365700	0.63514700	0.38665700

C	2.32829200	1.54514900	-1.69461700

C	2.84312800	0.22057000	-2.16842800

C	3.02098500	-2.22739600	-1.77507400

C	1.25334700	2.96795600	0.12282000

C	-0.26364800	2.85826300	0.35029100

C	1.98743600	3.25663100	1.44198900

O	1.52914100	3.99991600	-0.82914200

O	5.05007600	0.00513300	-0.49391400

H	-4.14335000	-0.09065800	-3.25580000

H	-1.79527300	-0.75894500	-2.55756200

H	-2.23286900	0.91126200	-2.21230300

H	-1.56212200	-0.60656200	3.35801800

H	-3.65698600	-2.50384200	-2.92262300

H	-5.29129900	-2.10771100	-2.34717100

H	-3.97177500	-2.34803300	-1.18199200

H	-4.74711500	0.42756400	2.20008100

H	0.25808900	-1.82896300	-1.44313400

H	0.24838000	-0.09514300	-1.71706700

H	4.26487400	-2.53229600	0.88262500

H	4.98438500	-1.15801100	1.71632200

H	2.75526400	-0.28870000	2.32873500

H	2.82674500	-1.98262100	2.81674800

H	2.18309500	-3.57006700	0.42528800

H	1.14972000	-3.53056100	1.85906600

H	0.44885900	-3.25124000	0.26139500

H	2.41514200	2.41540100	-2.33183500

H	2.24661900	-0.14404700	-3.01795900

H	3.86892900	0.33053700	-2.53565700

H	3.29448800	-3.04514800	-1.10758000

H	2.10232400	-2.50950100	-2.29724300

H	3.81542200	-2.13921200	-2.52308600

H	-0.64448500	3.78815400	0.79122500

H	-0.77144300	2.69480600	-0.60522900

H	-0.50909700	2.03532400	1.02520700

H	1.83435800	2.44707300	2.16061800

H	1.60784500	4.18478800	1.88740600

H	3.05911800	3.36915200	1.25393700

H	1.19731600	4.82668200	-0.44700500

**Table S3**. Important thermodynamic parameters and Boltzmann distributions of the optimized **3** at b3lyp/6-31g* level in gas phase.

| Conformations | Energy (a.u) | Δ G(kcal/mol) | % |
| --- | --- | --- | --- |
| 1 | -1997.092568 | 0 | 86.58% |
| 2 | -1997.090681 | 1.18 | 11.73% |
| 3 | -1997.088377 | 2.63 | 1.02% |
| 4 | -1997.087632 | 3.1 | 0.46% |
| 5 | -1997.086822 | 3.61 | 0.20% |

**Table S4**. The coordinates for the lowest energy conformers of **3**.

Energy = -1997.09256800 a.u. #Cluster: 1

C	-1.95023600	-1.32070400	1.19200800

O	4.83610800	-1.63394700	-0.57048500

C	3.65682900	-2.47153000	-0.65999500

C	2.52823900	-1.73154100	-1.36273800

C	2.26062000	-0.40653800	-0.69328500

C	3.35905300	0.22210200	-0.07218300

C	4.69659700	-0.37768200	-0.09988100

C	1.00012800	0.18486200	-0.69746800

C	0.84757000	1.44293500	-0.07225500

C	1.92790700	2.07535400	0.55586900

C	3.19922200	1.48901900	0.55175100

O	5.71191500	0.21114600	0.26946500

C	4.08978300	-3.74337000	-1.36441500

O	4.21312200	2.14292100	1.12120400

C	-0.20359400	-0.50191600	-1.30099300

C	-1.51934400	0.12684300	-0.82020000

C	-1.38290000	1.67742000	-0.93482900

O	-0.33051200	2.09483100	-0.02956300

C	-2.78112000	-0.46585500	-1.52031300

C	-4.02682900	0.35305100	-1.11439700

C	-3.90059500	1.86789000	-1.10705400

C	-2.63606400	2.36999200	-0.40113100

C	-0.99577400	2.22358700	-2.31517600

O	-1.68111700	-0.04526200	0.60664400

O	-0.80457800	-2.16826300	1.11398900

C	-3.05244500	-2.07354500	0.44647200

C	-2.94737700	-1.93140200	-1.06733100

C	-2.75033800	-0.47329000	-3.06655700

Cl	1.70390100	3.62915600	1.30891400

O	-5.09105800	-0.18900200	-0.88275400

C	-2.32415900	-1.00537300	2.66155900

C	-3.45902500	0.01737500	2.79607100

C	-2.64890700	-2.29331000	3.43059100

C	0.33846300	-1.78425600	1.87112100

H	3.34783700	-2.69768300	0.37004200

H	2.80020400	-1.57495200	-2.41706200

H	1.63867800	-2.36796600	-1.35892100

H	4.41275900	-3.52278900	-2.38704100

H	3.25703900	-4.45327100	-1.40429000

H	4.92277100	-4.21028600	-0.83165500

H	5.03097300	1.60678300	0.93807600

H	-0.15775400	-0.45882800	-2.39508500

H	-0.20379100	-1.55509300	-1.02342700

H	-3.90419200	2.19977500	-2.15506900

H	-4.80368300	2.27353400	-0.64449600

H	-2.70042100	2.16950800	0.66891700

H	-2.53143900	3.45257100	-0.52728400

H	-0.15522300	1.68681500	-2.75973800

H	-1.82664100	2.20633300	-3.02108200

H	-0.69344300	3.26562400	-2.17821500

H	-4.02441200	-1.69261000	0.76181900

H	-2.99984400	-3.12673600	0.73230500

H	-2.10519900	-2.51636000	-1.45174700

H	-3.85453600	-2.33252000	-1.52707800

H	-1.91992300	-1.08278500	-3.43467600

H	-3.67819100	-0.92640300	-3.43013400

H	-2.66665000	0.51667800	-3.51489000

H	-1.43526200	-0.54712100	3.11000700

H	-4.37843100	-0.31683100	2.30363100

H	-3.69171400	0.17125200	3.85565900

H	-3.17603300	0.98173700	2.37028500

H	-1.87803700	-3.05638500	3.28828000

H	-3.60502200	-2.71744500	3.10428800

H	-2.73178100	-2.08200300	4.50184400

H	0.24965500	-2.09681200	2.91904900

H	1.18993000	-2.30372800	1.42445600

H	0.52019300	-0.70774500	1.82225600

Energy = -1997.09068100 a.u. #Cluster: 2

C	-2.06739200	-1.02515400	1.40794100

O	4.79683600	-1.96346000	-0.09266900

C	3.90073200	-2.44328100	-1.12925200

C	2.44733000	-2.17686900	-0.74682800

C	2.24169600	-0.72358500	-0.39993500

C	3.32959400	-0.05168500	0.19063600

C	4.60767800	-0.73119800	0.42109900

C	1.02306300	-0.08499200	-0.59863000

C	0.90732800	1.27370600	-0.23164000

C	1.97191700	1.94859700	0.37936100

C	3.18770200	1.29536200	0.62020200

O	5.52392800	-0.24137500	1.08096300

C	4.30490300	-1.84167700	-2.47146400

O	4.17030300	1.96229800	1.22799000

C	-0.18617000	-0.83304800	-1.10949900

C	-1.48385200	-0.05870700	-0.83999500

C	-1.25674300	1.42517600	-1.26949100

O	-0.22425800	1.98080900	-0.41548700

C	-2.74379400	-0.73326500	-1.46346100

C	-3.95958100	0.20193400	-1.28718200

C	-3.75658100	1.67631200	-1.59896000

C	-2.49379200	2.26846600	-0.96146700

C	-0.77837500	1.64765000	-2.70968300

O	-1.72030500	0.08584000	0.57851500

O	-0.94822400	-1.89042500	1.58765500

C	-3.15500800	-1.89687000	0.77683800

C	-2.99111200	-2.06740500	-0.72952700

C	-2.65905700	-1.05715300	-2.97332600

Cl	1.78483800	3.61317400	0.85469900

O	-5.05491700	-0.23231300	-0.98483000

C	-2.51296800	-0.38594800	2.74675500

C	-3.62672100	0.65569600	2.58552000

C	-2.91282200	-1.46646300	3.76053100

C	0.16481300	-1.36547600	2.30645200

H	4.08549200	-3.52018100	-1.14102100

H	1.80324900	-2.48493500	-1.57564500

H	2.17530000	-2.80566400	0.11274000

H	4.11512800	-0.76423500	-2.49497900

H	3.73324900	-2.31027800	-3.27999800

H	5.36944700	-2.01235000	-2.65352200

H	4.91646400	1.31447100	1.34615000

H	-0.09607400	-1.02148000	-2.18568000

H	-0.24579500	-1.80255900	-0.61629600

H	-3.70641800	1.77349500	-2.69296900

H	-4.65326700	2.21037200	-1.27521000

H	-2.60676300	2.31220000	0.12231800

H	-2.33097100	3.29153000	-1.31607800

H	0.05703500	0.99820600	-2.97949400

H	-1.57410300	1.50759000	-3.44176000

H	-0.43473200	2.68338600	-2.78134900

H	-4.12904900	-1.44476700	0.96634300

H	-3.13970700	-2.87111100	1.27122200

H	-2.16422100	-2.74966900	-0.95309800

H	-3.89970000	-2.51972300	-1.13613900

H	-1.83834900	-1.75109800	-3.17697100

H	-3.58901100	-1.54909200	-3.27598400

H	-2.52641200	-0.18196400	-3.60972200

H	-1.63767100	0.14804400	3.13382800

H	-3.89499800	1.06190300	3.56711600

H	-3.30488400	1.48567000	1.95392200

H	-4.53506700	0.22753000	2.14855200

H	-3.05213100	-1.02117900	4.75122400

H	-2.15468800	-2.25122600	3.83830500

H	-3.85885900	-1.94181700	3.47802900

H	0.37580700	-0.32881000	2.03255100

H	0.01085100	-1.43568900	3.39037500

H	1.02497200	-1.98228000	2.03627000

Energy = -1997.08837700 a.u. #Cluster: 3

C	-1.98625900	-1.26165800	1.30188300

O	4.76716400	-1.63896800	-0.66548800

C	3.59029600	-2.48385300	-0.70239700

C	2.44363300	-1.76979100	-1.40263900

C	2.18311700	-0.42793200	-0.76471000

C	3.29198300	0.22411200	-0.18820800

C	4.63196600	-0.36982600	-0.22942800

C	0.91838000	0.15453700	-0.75276300

C	0.77376700	1.43025400	-0.16207500

C	1.86422900	2.08510600	0.42379000

C	3.13885900	1.50631500	0.40532000

O	5.65210700	0.23445200	0.09894600

C	4.01336200	-3.77553000	-1.37597800

O	4.16148200	2.18075500	0.93356700

C	-0.29660000	-0.56068700	-1.29929100

C	-1.60191800	0.07768700	-0.80391700

C	-1.47962000	1.62456700	-0.97398600

O	-0.40688600	2.07842900	-0.11046100

C	-2.87995400	-0.54763800	-1.44411200

C	-4.12734400	0.29997400	-1.10089100

C	-3.99237400	1.81344500	-1.12595500

C	-2.72588200	2.31977000	-0.42847400

C	-1.13138400	2.12477600	-2.38107800

O	-1.70411500	-0.03475100	0.63122100

O	-0.89847800	-2.17354800	1.21318400

C	-3.15231600	-2.00024900	0.63596400

C	-3.05973900	-1.97459800	-0.88496000

C	-2.85958000	-0.66427500	-2.98703100

Cl	1.64713700	3.65577700	1.14262700

O	-5.20398500	-0.22782000	-0.89530300

C	-2.36365600	-0.81870200	2.74402900

C	-2.58871000	-2.02678200	3.66158200

C	-1.42702400	0.21518400	3.39385900

C	0.33294800	-1.82797900	1.83582300

H	3.30472700	-2.67842600	0.34071400

H	2.69357200	-1.64134900	-2.46612200

H	1.55767600	-2.40991200	-1.36362700

H	4.86016000	-4.22197200	-0.84750200

H	4.31232000	-3.58638400	-2.41206600

H	3.18341600	-4.48983300	-1.37456300

H	4.97816300	1.64451300	0.74593500

H	-0.27900500	-0.55809300	-2.39473600

H	-0.28546400	-1.60393300	-0.98339700

H	-3.99401000	2.12315800	-2.18082100

H	-4.89480200	2.23329000	-0.67468400

H	-2.78311600	2.11675200	0.64245500

H	-2.62175400	3.40200300	-0.55672100

H	-0.27376500	1.60091000	-2.80831900

H	-1.96663200	2.03956400	-3.07671400

H	-0.86937900	3.18324000	-2.29694400

H	-4.09093300	-1.53273400	0.94199800

H	-3.16068600	-3.03201300	0.99487600

H	-2.22664700	-2.59786200	-1.22655300

H	-3.97570700	-2.39557200	-1.30737700

H	-2.05525800	-1.32924700	-3.31436300

H	-3.80634300	-1.10576700	-3.31373700

H	-2.74116500	0.28881900	-3.50356000

H	-3.32833400	-0.31200700	2.61221500

H	-2.89135700	-1.68819300	4.65826200

H	-3.37182100	-2.69129200	3.28412200

H	-1.67462900	-2.61893300	3.77183600

H	-1.94446600	0.68201200	4.23957900

H	-1.14765200	1.00116000	2.68880400

H	-0.51308700	-0.23587000	3.78892100

H	0.57206500	-0.76988700	1.71753700

H	0.32866700	-2.08805200	2.90059800

H	1.10452200	-2.42206000	1.33784600

Energy = -1997.08763200 a.u. #Cluster: 4

C	-1.71101100	-0.87827200	1.63481400

O	4.66234800	-1.88718300	-0.05210800

C	3.75970000	-2.50960300	-1.00253700

C	2.30865700	-2.20069500	-0.64710700

C	2.09309600	-0.71596400	-0.50029600

C	3.18179200	0.03576600	-0.01415800

C	4.46711600	-0.59686800	0.29078300

C	0.86877700	-0.11894300	-0.77159200

C	0.73618400	1.27055600	-0.55377400

C	1.80276600	2.02807600	-0.05519200

C	3.03178300	1.42567800	0.23864200

O	5.38805200	-0.01690800	0.86651800

C	4.14723200	-2.09644700	-2.41919600

O	4.01606900	2.17521000	0.73981800

C	-0.34181900	-0.92312100	-1.18596400

C	-1.61790200	-0.18252200	-0.76530000

C	-1.53717700	1.25578800	-1.37558600

O	-0.41523200	1.94084900	-0.76615600

C	-2.93747000	-0.96031100	-1.06350600

C	-4.16932000	-0.03622300	-0.91201800

C	-4.05538500	1.39572500	-1.40469400

C	-2.75713600	2.07700400	-0.96130200

C	-1.29868300	1.33464000	-2.88765800

O	-1.57583300	0.11654800	0.64513000

O	-0.57139300	-1.75061400	1.49585000

C	-2.99498800	-1.67203800	1.39169100

C	-3.06730600	-2.13309500	-0.06230700

C	-3.02701200	-1.56157900	-2.48560100

Cl	1.59175500	3.73186400	0.24249000

O	-5.22090900	-0.46562500	-0.47593800

C	-1.65008900	-0.11060200	2.97535800

C	-0.31371100	0.63587400	3.10759200

C	-2.82287700	0.86135400	3.16075300

C	-0.34131800	-2.70997600	2.50959100

H	3.95172600	-3.57719700	-0.86865900

H	1.66184700	-2.62532100	-1.41993400

H	2.04512600	-2.69786500	0.29649900

H	5.21054800	-2.28744700	-2.58802700

H	3.95268300	-1.03281000	-2.58740900

H	3.56881100	-2.67248400	-3.14984100

H	4.76896400	1.55370600	0.93203500

H	-0.35809700	-1.08843400	-2.26919900

H	-0.31879200	-1.89871700	-0.70791000

H	-4.11451000	1.37360500	-2.50216000

H	-4.93630200	1.93758400	-1.05167200

H	-2.74120000	2.18363000	0.12483900

H	-2.68122700	3.08044900	-1.39195000

H	-0.38540800	0.81382100	-3.18250500

H	-2.12751700	0.93419500	-3.47269200

H	-1.17616400	2.38942700	-3.14959100

H	-3.85856900	-1.04212200	1.60931700

H	-3.05143400	-2.52930500	2.07040200

H	-2.27890300	-2.86517900	-0.26585800

H	-4.02685000	-2.62368900	-0.24126800

H	-2.21052800	-2.26860300	-2.65780900

H	-3.96957300	-2.11047900	-2.57566800

H	-3.00094300	-0.81489000	-3.28100900

H	-1.71784200	-0.86700900	3.76882400

H	-0.26229600	1.45512800	2.38521500

H	-0.21458200	1.05780700	4.11329700

H	0.54009900	-0.01974600	2.91908700

H	-3.79163500	0.35424100	3.19616400

H	-2.84729100	1.59452300	2.34919200

H	-2.70355300	1.40827300	4.10209600

H	0.01269800	-2.25218800	3.44175600

H	-1.22963200	-3.31687700	2.72923600

H	0.44231800	-3.37113300	2.12932300

Energy = -1997.08682200 a.u. #Cluster: 5

C	-2.11821800	0.19767600	1.75310000

O	4.73704400	-1.66479100	1.09752000

C	3.81755300	-2.68456300	0.62598200

C	2.37345000	-2.21480900	0.77979000

C	2.17303500	-0.87398200	0.11945600

C	3.27287600	0.00534400	0.12048300

C	4.55753500	-0.38661900	0.70765900

C	0.94824500	-0.49497000	-0.41821500

C	0.84121100	0.78067000	-1.01502800

C	1.91665900	1.67765200	-0.99466100

C	3.13804900	1.31617000	-0.41107400

O	5.48743700	0.39999800	0.88390300

C	4.18710300	-3.08588800	-0.79852900

O	4.13213000	2.20555200	-0.38849000

C	-0.27264600	-1.37768500	-0.30049600

C	-1.56175000	-0.59309400	-0.57976000

C	-1.34110700	0.25219500	-1.87543400

O	-0.29219200	1.21656500	-1.59935300

C	-2.83836600	-1.48810300	-0.59086700

C	-4.05064700	-0.68400400	-1.11290000

C	-3.83109700	0.23989300	-2.29974600

C	-2.57402100	1.10646400	-2.16731300

C	-0.88732500	-0.52117400	-3.11883600

O	-1.74690400	0.45054300	0.39837200

O	-1.06331200	-0.41763900	2.47881200

C	-3.27516200	-0.80517300	1.83073900

C	-3.11296500	-1.96253000	0.85207300

C	-2.75634800	-2.75393400	-1.47728700

Cl	1.73615100	3.26208200	-1.69307200

O	-5.16031200	-0.84482200	-0.64112400

C	-2.56342000	1.59071200	2.28277900

C	-2.89228900	1.54036300	3.77987500

C	-1.62856400	2.76673600	1.94933400

C	0.13862000	0.31706200	2.68418400

H	4.00488200	-3.52234800	1.30199300

H	1.70908800	-2.97245800	0.35401700

H	2.12622300	-2.14422600	1.84835200

H	5.24710000	-3.34890800	-0.85091800

H	3.99486100	-2.26995300	-1.50188100

H	3.59651600	-3.95489000	-1.10862800

H	4.88166300	1.77439800	0.10400500

H	-0.20404100	-2.22328500	-0.99459400

H	-0.32706600	-1.79175000	0.70641100

H	-3.76240100	-0.39244700	-3.19659200

H	-4.72961900	0.85128600	-2.41414100

H	-2.69448600	1.81470600	-1.34571300

H	-2.40652200	1.68382500	-3.08228400

H	-0.03335900	-1.17039700	-2.91445000

H	-1.68425700	-1.12691700	-3.55133300

H	-0.57827000	0.21376000	-3.86747900

H	-4.20958100	-0.28572600	1.60757200

H	-3.33936400	-1.18376400	2.85358600

H	-2.29790100	-2.62108500	1.16988500

H	-4.02917300	-2.55876500	0.84810700

H	-1.97168300	-3.42726800	-1.12038300

H	-3.70814900	-3.28963300	-1.40637900

H	-2.56910000	-2.54601100	-2.53135100

H	-3.49751200	1.78446300	1.73990300

H	-3.23668800	2.52260400	4.12073300

H	-3.68074600	0.81534300	4.00357200

H	-2.01266300	1.26868700	4.37208700

H	-2.18269300	3.70497600	2.06556800

H	-1.26500500	2.71042200	0.92103800

H	-0.76541800	2.82146200	2.61787100

H	0.91798000	-0.42090100	2.89121300

H	0.42440600	0.89533700	1.80389700

H	0.05482100	0.98436900	3.54956400

**Table S5**. Important thermodynamic parameters and Boltzmann distributions of the optimized **4** at b3lyp/6-31g* level in gas phase.

| Conformations | Energy (a.u) | Δ G(kcal/mol) | % |
| --- | --- | --- | --- |
| 1 | -1765.342607 | 0 | 71.52% |
| 2 | -1765.34167 | 0.59 | 26.51% |
| 3 | -1765.338911 | 2.32 | 1.43% |
| 4 | -1765.337456 | 3.23 | 0.31% |
| 5 | -1765.337237 | 3.37 | 0.24% |

**Table S6**. The coordinates for the lowest energy conformers of **4**.

Energy = -1765.34260700 a.u. #Cluster: 1

C	2.21558400	-0.89451000	1.49881600

O	-4.77322600	-0.51186000	0.82368700

C	-3.75487100	0.09013800	1.65035400

C	-2.62731800	0.69361200	0.82651600

C	-2.11310300	-0.27907900	-0.20149500

C	-3.01239400	-1.26095400	-0.66367600

C	-4.39575800	-1.31923100	-0.19096500

C	-0.81274500	-0.18315000	-0.68686600

C	-0.41534400	-1.10856900	-1.67878200

C	-1.27741600	-2.09850400	-2.14440800

C	-2.58220200	-2.18041400	-1.65942800

O	-5.24898100	-2.05645000	-0.68230100

C	-4.45977600	1.08802600	2.54966500

O	-3.39089100	-3.12182200	-2.16487000

C	0.17039600	0.83124700	-0.15903900

C	1.61090900	0.46613600	-0.52810800

C	1.64197600	0.08681000	-2.04285700

O	0.82480400	-1.09976600	-2.22188500

C	2.63739500	1.56116700	-0.10485900

C	4.02555500	1.20527100	-0.67633300

C	4.08823100	0.72975200	-2.11995400

C	3.04747800	-0.34519700	-2.45678800

C	1.07948900	1.13562500	-3.01136000

O	2.00956700	-0.78044500	0.09004700

O	0.97357800	-0.83160000	2.19997400

C	3.04881000	0.26004900	2.05654300

C	2.69365800	1.60639700	1.43652400

C	2.31657600	2.99937500	-0.57755700

O	5.04104000	1.36597100	-0.02568400

C	2.89848000	-2.27247200	1.68184200

C	4.17640800	-2.43617600	0.84980100

C	0.05538500	-1.89715800	1.98484900

O	-3.10993800	1.87700000	0.13334300

C	-2.51970600	3.06142400	0.42900000

O	-1.61765400	3.19578600	1.22782000

C	-3.14397600	4.16986000	-0.38039800

C	3.16893100	-2.55680500	3.16534200

H	-3.30499500	-0.71192100	2.25234400

H	-1.84008600	1.00911900	1.51176300

H	-0.94328300	-2.78811500	-2.91033800

H	-5.21817400	0.58127100	3.15175200

H	-4.94944600	1.85538900	1.94504300

H	-3.73796000	1.56969700	3.21696000

H	-4.28644500	-2.97075400	-1.76398900

H	-0.06083500	1.82541700	-0.55075200

H	0.09302700	0.90283700	0.92348600

H	3.93808600	1.61121600	-2.75948300

H	5.10411500	0.37322900	-2.30728300

H	3.28322600	-1.27098500	-1.93059300

H	3.05866700	-0.56358200	-3.52996600

H	0.10892700	1.52173200	-2.69278000

H	1.75444900	1.97952600	-3.15642200

H	0.94384000	0.64522000	-3.97964500

H	4.10469900	0.06235600	1.86815500

H	2.90515300	0.29142400	3.13922400

H	1.73049000	1.96592800	1.81240500

H	3.44755300	2.34185800	1.72994900

H	1.36488900	3.33916900	-0.15830700

H	3.09883200	3.66808300	-0.20413300

H	2.27347300	3.11458300	-1.66098300

H	2.18420100	-3.01577600	1.30885900

H	4.60367800	-3.43043100	1.02232400

H	3.97001300	-2.33783900	-0.21757500

H	4.94176600	-1.69982000	1.11737200

H	0.29729700	-2.76915000	2.60575400

H	-0.92653600	-1.52082600	2.28301100

H	0.01141800	-2.19468800	0.93415300

H	-2.64254700	5.11174800	-0.15727800

H	-3.06544600	3.94267400	-1.44817800

H	-4.20987700	4.24902100	-0.14348100

H	3.48387300	-3.59709000	3.29931000

H	2.28140200	-2.38287400	3.78054500

H	3.97223700	-1.91818400	3.54945700

Energy = -1765.34167000 a.u. #Cluster: 2

C	1.70550200	-0.23286200	1.84885800

O	-4.64656900	-0.07691800	0.59285800

C	-3.62079300	0.73150000	1.20541400

C	-2.43750800	0.97280500	0.28095200

C	-1.96317000	-0.30541200	-0.35258500

C	-2.87793300	-1.37627500	-0.42788600

C	-4.27392500	-1.22064600	-0.02426300

C	-0.66010800	-0.41664700	-0.82111000

C	-0.24359000	-1.68204700	-1.29330100

C	-1.12049400	-2.76065600	-1.37240900

C	-2.44871300	-2.61574600	-0.97497000

O	-5.14233500	-2.06544400	-0.23778200

C	-4.29589400	2.00309700	1.68400300

O	-3.27516000	-3.66214300	-1.11723100

C	0.32350400	0.72398300	-0.78796700

C	1.73764000	0.16183600	-0.61776400

C	1.98012800	-0.84808100	-1.78822600

O	1.03553100	-1.94114800	-1.66272400

C	2.83709900	1.25611300	-0.44753900

C	4.25420800	0.65765500	-0.61592400

C	4.46331100	-0.44130800	-1.64453300

C	3.35460700	-1.49801600	-1.64068000

C	1.77863200	-0.29828300	-3.20569300

O	1.77437800	-0.71861000	0.52744100

O	0.39755400	0.35702900	2.00851100

C	2.79859300	0.81063600	2.06444500

C	2.72828900	1.87186900	0.96905500

C	2.73207100	2.42843600	-1.45202800

O	5.19584000	1.10490700	0.01214600

C	1.80217000	-1.49177800	2.74237700

C	0.65629800	-2.46416300	2.42598600

C	0.14423600	1.09374900	3.19029200

O	-2.78904000	1.91778500	-0.76609600

C	-2.17651000	3.12538300	-0.74109800

O	-1.37405700	3.47080600	0.10129700

C	-2.62675400	3.96377700	-1.91085700

C	3.15696100	-2.20361600	2.63626200

H	-3.22460800	0.16540600	2.06003600

H	-1.64443000	1.41877300	0.87828600

H	-0.77323100	-3.71198100	-1.75735500

H	-4.74418300	2.53024800	0.83781400

H	-3.56364100	2.66232500	2.16101900

H	-5.08422100	1.76411900	2.40228400

H	-4.17642100	-3.34906700	-0.84444400

H	0.27034300	1.30330200	-1.71639400

H	0.09751600	1.41501200	0.01675500

H	4.52207300	0.04256300	-2.62999500

H	5.44442000	-0.88455000	-1.45568500

H	3.36882600	-2.05387100	-0.70117300

H	3.50552800	-2.21696500	-2.45263900

H	0.77942600	0.12075100	-3.34106200

H	2.51101600	0.46149400	-3.48044800

H	1.88256900	-1.13569300	-3.90172400

H	3.77220300	0.32007900	2.02529700

H	2.71527700	1.26323700	3.05734400

H	1.79264200	2.43644700	1.04023400

H	3.55145600	2.57939000	1.08996500

H	1.77386500	2.94295400	-1.33529800

H	3.52799000	3.14738500	-1.23476700

H	2.83267500	2.12577400	-2.49567500

H	1.68940300	-1.14164500	3.77764900

H	0.64802100	-3.28719300	3.14877200

H	-0.31255900	-1.96023700	2.45714400

H	0.77822400	-2.88553300	1.42416100

H	0.64993700	2.06804800	3.19096100

H	-0.93511700	1.26756300	3.21549900

H	0.42623200	0.54510600	4.09787200

H	-2.13905000	4.93798100	-1.87193000

H	-2.37818500	3.45617000	-2.84850600

H	-3.71412300	4.08637700	-1.88752600

H	3.15386100	-3.10291900	3.26164700

H	3.99099200	-1.57718100	2.96680100

H	3.34899700	-2.51560300	1.60484800

Energy = -1765.33891100 a.u. #Cluster: 3

C	-2.28426700	-0.96977800	-1.47834000

O	4.73999100	-0.45529700	-0.76844100

C	3.72685500	0.11453900	-1.62381700

C	2.58378300	0.72925000	-0.83017200

C	2.06452500	-0.22145500	0.21563600

C	2.96700000	-1.18111100	0.71607700

C	4.35728700	-1.23788000	0.26339300

C	0.75634400	-0.12608600	0.68040100

C	0.35671900	-1.02487800	1.69593600

C	1.22159900	-1.99381700	2.19838100

C	2.53284800	-2.07708600	1.73132700

O	5.21108500	-1.95224800	0.78626100

C	4.43430500	1.09440100	-2.54078500

O	3.34379400	-2.99615600	2.27275600

C	-0.22995700	0.86369100	0.11146200

C	-1.67035400	0.49905500	0.48103100

C	-1.71048400	0.16768100	2.00764400

O	-0.88874400	-1.00913500	2.22881400

C	-2.70271800	1.57026100	0.01269500

C	-4.08943400	1.28360800	0.62967900

C	-4.14479700	0.83011700	2.08027300

C	-3.11892300	-0.25781600	2.41707100

C	-1.15929000	1.24857800	2.94569300

O	-2.03999300	-0.77658800	-0.08663300

O	-1.08816200	-0.87490300	-2.24161500

C	-3.18269400	0.13564200	-2.04437000

C	-2.79923900	1.51658100	-1.52686800

C	-2.35442000	3.03162400	0.38668300

O	-5.11126800	1.48640600	0.00103500

C	-2.99969700	-2.34935600	-1.54353800

C	-3.23541200	-2.79091200	-2.99299200

C	-0.06313800	-1.83481500	-2.02219700

O	3.04769600	1.93390200	-0.16116000

C	2.45054300	3.10510500	-0.49362700

O	1.55684300	3.21056500	-1.30612900

C	3.05526200	4.23958300	0.29408200

C	-2.36354600	-3.47431700	-0.70813400

H	3.29186500	-0.70772200	-2.20916500

H	1.80293200	1.02147300	-1.53274500

H	0.88534300	-2.66347900	2.98092500

H	4.90672500	1.88393500	-1.95116400

H	3.71768000	1.54890100	-3.23235400

H	5.20695500	0.57916000	-3.11702300

H	4.24319500	-2.84710300	1.87992000

H	-0.00771100	1.87048700	0.47428900

H	-0.14588400	0.90575600	-0.97304000

H	-3.97399200	1.71817500	2.70551400

H	-5.16538600	0.49568600	2.28285700

H	-3.36244000	-1.17734600	1.88158100

H	-3.13445000	-0.48146600	3.48896700

H	-1.81818000	2.11353900	3.02655300

H	-1.06449600	0.80382600	3.94059300

H	-0.17061500	1.59739300	2.63981300

H	-4.21923500	-0.07276600	-1.76903500

H	-3.11591900	0.11209300	-3.13458400

H	-1.84176400	1.83336800	-1.95251400

H	-3.55247500	2.24110800	-1.84682400

H	-1.42569300	3.34289100	-0.10049700

H	-3.15463900	3.68252000	0.02039000

H	-2.25177400	3.20278700	1.45898700

H	-3.97895000	-2.15206300	-1.08876200

H	-3.77718300	-3.74278400	-3.01140200

H	-3.82636200	-2.06083100	-3.55415500

H	-2.28915400	-2.93268200	-3.52501800

H	-0.23770300	-2.74704900	-2.60486600

H	0.86672900	-1.37564800	-2.37063700

H	0.04512400	-2.08888000	-0.96641500

H	2.96833600	4.03816900	1.36635900

H	4.12262000	4.32397900	0.06559900

H	2.54655100	5.17034000	0.04254100

H	-2.04944600	-3.11208800	0.27316000

H	-1.50024600	-3.92834000	-1.20202100

H	-3.10251500	-4.27015700	-0.56066800

Energy = -1765.33745600 a.u. #Cluster: 4

C	2.29038100	-0.73411300	1.61425100

O	-4.82564400	-0.38284500	0.77776900

C	-3.80406800	0.23826300	1.58533600

C	-2.63222000	0.73862500	0.75466600

C	-2.14017800	-0.31647500	-0.19986500

C	-3.06291000	-1.30093900	-0.60793900

C	-4.45569200	-1.27950200	-0.16219200

C	-0.83427400	-0.28769600	-0.67958300

C	-0.45283200	-1.28696100	-1.60075500

C	-1.33780200	-2.28218600	-2.01015100

C	-2.64942600	-2.29401800	-1.53845600

O	-5.32423000	-2.02339500	-0.61538800

C	-4.48963600	1.32267500	2.39513700

O	-3.48057800	-3.24219500	-1.99259100

C	0.15591800	0.77038600	-0.27400400

C	1.60327000	0.34931000	-0.55416500

C	1.69855400	-0.22478700	-1.99536300

O	0.79034500	-1.35148300	-2.12829800

C	2.59889100	1.53266500	-0.31886500

C	3.93361700	1.29108500	-1.05735400

C	4.24646600	-0.09998300	-1.57499600

C	3.08209700	-0.84289300	-2.26111400

C	1.33772700	0.76856700	-3.10424400

O	1.94554100	-0.81773100	0.23548900

O	1.17773100	-0.32397400	2.40730200

C	3.34403300	0.34658300	1.84536300

C	2.91662000	1.65561800	1.19787000

C	2.06744000	2.92245800	-0.73882500

O	4.74633200	2.19182400	-1.16247700

C	2.75534500	-2.15995100	1.99918100

C	3.88742300	-2.69873000	1.11820800

C	0.07043800	-1.21348700	2.47604600

O	-3.02789700	1.90326100	-0.01877700

C	-2.40821100	3.07699200	0.26333800

O	-1.56828900	3.21508800	1.12676100

C	-2.90429700	4.16309200	-0.65666200

C	3.13619000	-2.23523400	3.48383600

H	-3.39971300	-0.53562300	2.25316100

H	-1.84782800	1.05512700	1.44253500

H	-1.01528900	-3.03030900	-2.72434700

H	-5.27945700	0.88787400	3.01283200

H	-4.93713400	2.06278300	1.72686200

H	-3.76443500	1.82489700	3.04327300

H	-4.37578200	-3.03824800	-1.61507900

H	-0.06071300	1.69334000	-0.81734300

H	0.06857100	1.01860200	0.78293500

H	5.11985700	-0.01480100	-2.22565500

H	4.55799700	-0.67552200	-0.69611700

H	3.04716400	-1.87658800	-1.91689900

H	3.24301700	-0.87219700	-3.34333000

H	0.39242300	1.28404900	-2.91736300

H	2.12301600	1.51567200	-3.24760700

H	1.23710400	0.20463600	-4.03585300

H	4.30327000	0.01090300	1.44048500

H	3.47571400	0.48735400	2.92102400

H	2.02301900	2.02984600	1.70687000

H	3.69871900	2.41281900	1.31020100

H	1.24559700	3.23793800	-0.09109400

H	2.88601700	3.63741200	-0.63431700

H	1.72567900	2.96222700	-1.77417400

H	1.89024100	-2.81083600	1.82646400

H	4.12508600	-3.72783800	1.40943700

H	3.60047200	-2.70307200	0.06490300

H	4.80707000	-2.11286600	1.23056600

H	-0.77562900	-0.61720100	2.82886700

H	-0.17333100	-1.63644000	1.49781500

H	0.24364500	-2.02397600	3.19512800

H	-2.66173700	3.90762300	-1.69347600

H	-3.99337100	4.24583800	-0.58945500

H	-2.43682200	5.11047400	-0.38790600

H	3.27667100	-3.27863600	3.78529600

H	2.36916600	-1.79070300	4.12409400

H	4.07804800	-1.70913600	3.67673800

Energy = -1765.33723700 a.u. #Cluster: 5

C	2.05843800	-0.47201500	1.65699800

O	-4.86466600	-0.00915900	0.83890700

C	-4.23407300	1.19395500	0.35211800

C	-2.71010900	1.06269600	0.46645500

C	-2.20086700	-0.18165500	-0.22536300

C	-3.10217700	-1.27227000	-0.25139300

C	-4.42955000	-1.19608500	0.36223400

C	-0.90043400	-0.29949700	-0.71287600

C	-0.53965500	-1.53008400	-1.31435400

C	-1.40872900	-2.61515100	-1.35943700

C	-2.68124000	-2.51386100	-0.80392100

O	-5.16767000	-2.16938900	0.50618000

C	-4.73272800	1.51362500	-1.05272300

O	-3.46709100	-3.59812300	-0.82187700

C	0.15498900	0.76647900	-0.51870900

C	1.56891800	0.20789900	-0.71365900

C	1.58174100	-0.62869900	-2.03033000

O	0.68432100	-1.75129300	-1.84769600

C	2.67250800	1.30773300	-0.63034500

C	4.03519700	0.68487300	-1.00159600

C	4.07707400	-0.22414200	-2.21988600

C	2.95378200	-1.26783400	-2.24614700

C	1.10242500	0.09503700	-3.29533000

O	1.88322200	-0.79921200	0.27594500

O	0.81398800	-0.12314500	2.25782400

C	2.95491000	0.75310200	1.84920600

C	2.71170300	1.84656200	0.81467400

C	2.48656300	2.52775000	-1.56246600

O	5.05020300	0.96861100	-0.39416300

C	2.65449800	-1.75464700	2.28754300

C	3.93109300	-2.24295000	1.59152500

C	-0.16397700	-1.15299500	2.37217700

O	-2.13150700	2.24607600	-0.12283300

C	-1.91875300	3.29583600	0.71579100

O	-2.24836900	3.30376900	1.87918100

C	-1.18981300	4.40504200	0.00117100

C	2.88872100	-1.56651900	3.79264400

H	-4.55905900	1.96665300	1.05129100

H	-2.43874200	1.04795600	1.52768500

H	-1.08401700	-3.54909700	-1.80183400

H	-5.82554000	1.54903000	-1.04876300

H	-4.40958500	0.75437300	-1.77118500

H	-4.34970200	2.48294600	-1.38149000

H	-4.29534600	-3.35728800	-0.32929100

H	-0.00329800	1.59437300	-1.21259900

H	0.07690000	1.17163200	0.48804500

H	4.01248800	0.42000500	-3.10857700

H	5.06138400	-0.69816300	-2.24548800

H	3.10817500	-2.00196100	-1.45428500

H	2.95684000	-1.80642300	-3.19978600

H	0.15892200	0.62386400	-3.14396200

H	1.83678700	0.80374100	-3.67937400

H	0.93936900	-0.66654500	-4.06332500

H	3.99877100	0.44599700	1.77989900

H	2.78842600	1.14124600	2.85670800

H	1.77235500	2.37093000	1.02117800

H	3.51422900	2.58583200	0.88484500

H	3.29459600	3.24022700	-1.36800400

H	2.51014800	2.28380000	-2.62451000

H	1.54005600	3.03628700	-1.35723700

H	1.90098000	-2.53820600	2.14697800

H	4.29095800	-3.15472900	2.08121400

H	3.74766500	-2.47368100	0.54041400

H	4.73807300	-1.50417900	1.64139200

H	-0.21528900	-1.77039700	1.47190600

H	0.02462100	-1.78979200	3.24526700

H	-1.12354700	-0.65035200	2.51108400

H	-1.34654500	5.34534400	0.53109000

H	-0.11671900	4.17914000	0.00168700

H	-1.51597600	4.48734600	-1.03830200

H	3.72789900	-0.88710300	3.97965300

H	3.13383100	-2.52655600	4.25909400

H	2.00772500	-1.15444500	4.29283600

**Table S7**. Important thermodynamic parameters and Boltzmann distributions of the optimized **5** at b3lyp/6-31g* level in gas phase.

| Conformations | Energy (a.u) | Δ G(kcal/mol) | % |
| --- | --- | --- | --- |
| 1 | -2260.882499 | 0 | 48.61% |
| 2 | -2260.882016 | 0.3 | 29.14% |
| 3 | -2260.881669 | 0.52 | 20.18% |
| 4 | -2260.879321 | 1.99 | 1.68% |
| 5 | -2260.877945 | 2.86 | 0.39% |

**Table S8**. The coordinates for the lowest energy conformers of **5**.

Energy = -22**6**0.88249900 a.u. #Cluster: 1

C	-2.91202900	-0.09665700	1.32143000

O	4.91482900	-0.33875100	1.17808200

C	3.86515900	-1.21238200	1.64725700

C	2.72203500	-1.32901800	0.64974300

C	2.26790000	0.02182100	0.16673800

C	3.19745500	1.07804800	0.22323300

C	4.57864300	0.86098200	0.66393600

C	0.98468200	0.20411600	-0.33977400

C	0.61470600	1.49786700	-0.77169100

C	1.51919900	2.56557900	-0.69947100

C	2.82562400	2.37163800	-0.23134400

O	5.46177200	1.71102800	0.56538400

C	4.52237100	-2.53768200	1.98369200

O	3.67183800	3.40236300	-0.23812000

C	-0.00935000	-0.92991200	-0.43952700

C	-1.43423300	-0.40439000	-0.58870700

C	-1.45874100	0.68972400	-1.70098700

O	-0.61227500	1.78354400	-1.24799100

C	-2.51305400	-1.51803500	-0.74467300

C	-3.86468300	-0.75814200	-0.77755000

C	-3.95404000	0.25385800	-1.92441700

C	-2.85195300	1.31322800	-1.82567200

C	-0.90870800	0.27173800	-3.06988000

O	-1.72263400	0.30413600	0.64050900

O	-4.01724800	0.00109100	0.43627500

C	-2.74639900	-1.54038200	1.78263600

C	-2.50069800	-2.40027400	0.52762100

C	-2.35039500	-2.44653300	-1.95428900

Cl	1.03469200	4.14480300	-1.24465100

O	-4.89205600	-1.71563000	-0.85428400

C	-3.08320900	0.94666900	2.45592500

C	-3.26902000	2.35478700	1.87678900

C	-4.24179100	0.58432200	3.38050600

O	-1.89545600	0.89909300	3.25211900

O	3.13533400	-2.11345500	-0.50069300

C	2.52969400	-3.31467800	-0.68297700

O	1.67307200	-3.76421400	0.04762700

C	3.07263200	-3.99041100	-1.91606500

H	3.44086500	-0.75654600	2.55215800

H	1.91274700	-1.87047500	1.14074200

H	5.28549900	-2.39450000	2.75272900

H	4.99887800	-2.95565500	1.09326500

H	3.77383300	-3.24699400	2.35032200

H	4.55473000	3.04320800	0.04647400

H	0.24289200	-1.58779300	-1.27770900

H	0.03517800	-1.55189200	0.45502200

H	-3.92352200	-0.28237000	-2.87833100

H	-4.93315300	0.74458100	-1.86622500

H	-3.02398300	1.92608100	-0.94089700

H	-2.87226600	1.97877300	-2.69495000

H	-0.73290700	1.18497600	-3.64543300

H	0.03916600	-0.26641500	-2.99499300

H	-1.61266300	-0.34620600	-3.62758400

H	-1.91925700	-1.58217500	2.49451300

H	-3.65324800	-1.86070200	2.29990600

H	-3.28852700	-3.14693200	0.41502600

H	-1.55290800	-2.94316500	0.58403100

H	-3.12292200	-3.21742300	-1.90827800

H	-2.45008900	-1.94444800	-2.91576500

H	-1.37624500	-2.94436400	-1.92885700

H	-5.72556000	-1.24293700	-0.69762400

H	-3.30436100	3.07324200	2.70096900

H	-2.43417500	2.61562600	1.22049200

H	-4.19645000	2.42723900	1.30187600

H	-4.37936700	1.37831500	4.12038800

H	-4.03766800	-0.34479900	3.91874500

H	-5.16660200	0.47091600	2.80790900

H	-1.16198900	1.05757000	2.63324100

H	2.54930500	-4.93319500	-2.07622200

H	2.95166700	-3.33694400	-2.78565500

H	4.14512500	-4.17517100	-1.79637600

Energy = -2260.88201600 a.u. #Cluster: 2

C	-2.93581000	-0.06282700	1.32109300

O	4.94160900	-0.27681500	1.16805800

C	3.90683500	-1.15137000	1.66674700

C	2.75411700	-1.30076300	0.68490500

C	2.28103000	0.03468300	0.17803100

C	3.19980800	1.10154000	0.20088300

C	4.58732400	0.90876100	0.63274700

C	0.99090600	0.19253600	-0.31825400

C	0.60420700	1.47106000	-0.77854100

C	1.49716700	2.54962100	-0.74031500

C	2.80971500	2.38047400	-0.27876300

O	5.46026600	1.76574500	0.50854000

C	4.58215700	-2.46233000	2.02340600

O	3.64438900	3.41987600	-0.31672300

C	0.00757000	-0.95322400	-0.37918900

C	-1.42286300	-0.45062500	-0.54792800

C	-1.45621000	0.61243800	-1.69128600

O	-0.63233500	1.73051800	-1.25029900

C	-2.48373100	-1.58614900	-0.66594100

C	-3.84004700	-0.84819800	-0.76754800

C	-3.92461900	0.11268800	-1.95729700

C	-2.85822300	1.20645100	-1.84998300

C	-0.88306900	0.16890100	-3.04201000

O	-1.72225300	0.29164100	0.65738900

O	-4.01685200	-0.03748000	0.40193500

C	-2.77886900	-1.47775500	1.86594900

C	-2.50182400	-2.39774200	0.65532600

C	-2.27540400	-2.57494200	-1.81859500

Cl	0.99110100	4.10986100	-1.31825700

O	-4.85602800	-1.82112600	-0.81636300

C	-3.16369100	1.08674400	2.33874800

C	-4.56673000	1.03237500	2.93624600

C	-2.08976700	1.05904000	3.43669500

O	-3.07892600	2.32403900	1.63060700

O	3.16332500	-2.10579800	-0.45295900

C	2.56422500	-3.31409800	-0.60682700

O	1.71919000	-3.75654900	0.14156500

C	3.09464100	-4.00623000	-1.83622700

H	3.48742200	-0.68123600	2.56666600

H	1.95532400	-1.83952800	1.19555500

H	5.35201400	-2.29491800	2.78076000

H	5.05358800	-2.89369900	1.13667900

H	3.84557200	-3.17203700	2.41284200

H	4.53354300	3.07743100	-0.03154800

H	0.26617400	-1.63481600	-1.19593100

H	0.05874800	-1.54607300	0.53500700

H	-3.84644300	-0.45493300	-2.89029300

H	-4.91841000	0.57584500	-1.94587000

H	-3.07645500	1.81845500	-0.97338100

H	-2.87518200	1.86188800	-2.72678000

H	-0.72092200	1.06865100	-3.64229000

H	0.07445000	-0.34776000	-2.94331800

H	-1.57006000	-0.47883800	-3.58744900

H	-1.95459600	-1.50459500	2.58193300

H	-3.69293400	-1.77304500	2.38777500

H	-3.28354600	-3.15294400	0.56138000

H	-1.55314100	-2.93020700	0.76644000

H	-2.30722700	-2.11472700	-2.80573600

H	-1.31598700	-3.09090400	-1.71394100

H	-3.06728600	-3.32654900	-1.78243800

H	-5.69694200	-1.34958900	-0.70097900

H	-5.31489100	1.08687300	2.14379000

H	-4.71905500	0.11278000	3.51014400

H	-4.70103700	1.88557800	3.60770200

H	-1.08784300	1.04323300	2.99627400

H	-2.19317900	0.19199000	4.09759400

H	-2.18948500	1.96578400	4.03970000

H	-2.26164700	2.27314400	1.10401600

H	2.92600700	-3.38056800	-2.71847900

H	4.17563100	-4.15256200	-1.74494800

H	2.59689600	-4.96851500	-1.95694500

Energy = -2260.88166900 a.u. #Cluster: 3

C	-2.85844200	-0.12323100	1.33118300

O	4.92286600	-0.38801200	1.15725700

C	3.86571000	-1.25529600	1.62040400

C	2.71445900	-1.34490200	0.62918400

C	2.27646000	0.01675100	0.16256200

C	3.21734500	1.06237100	0.23280700

C	4.59716300	0.82422900	0.66501700

C	0.99557800	0.21916300	-0.34115700

C	0.63775200	1.52256800	-0.75241600

C	1.55322700	2.57983600	-0.66597500

C	2.85795500	2.36606600	-0.20252700

O	5.48925600	1.66621200	0.57760600

C	4.50895100	-2.59276800	1.93484000

O	3.71488400	3.38822200	-0.19592700

C	-0.00795700	-0.90354900	-0.46252600

C	-1.42866400	-0.36511900	-0.60289300

C	-1.45024000	0.75386400	-1.68769500

O	-0.58580700	1.82804400	-1.22343800

C	-2.51078600	-1.47367700	-0.79020500

C	-3.86100300	-0.71548100	-0.78591200

C	-3.95410300	0.35313700	-1.88130000

C	-2.83841700	1.39574300	-1.76571400

C	-0.92595400	0.36355600	-3.07404200

O	-1.71644500	0.33303200	0.63584800

O	-4.00647100	-0.03593400	0.47347200

C	-2.65381000	-1.57755600	1.74141200

C	-2.50048500	-2.40197200	0.44941600

C	-2.35916300	-2.35994300	-2.03292600

Cl	1.08408500	4.17181300	-1.18761100

O	-4.88806400	-1.66650800	-0.91782900

C	-3.13203700	0.86290900	2.49518000

C	-2.03304400	0.79086100	3.55239600

C	-3.27763800	2.29695100	1.97074200

O	-4.34112400	0.44336900	3.13450500

O	3.10629900	-2.12517500	-0.53176200

C	2.48644500	-3.31869900	-0.71546500

O	1.63712800	-3.76732000	0.02431200

C	3.00145300	-3.98638400	-1.96487200

H	3.45211100	-0.80681100	2.53394800

H	1.90066400	-1.87852100	1.12141700

H	3.75442600	-3.29813900	2.29672800

H	5.27887700	-2.46897200	2.70047400

H	4.97449300	-3.00424000	1.03566600

H	4.59442700	3.01575800	0.08141600

H	0.23976700	-1.54527200	-1.31483000

H	0.03081500	-1.54606400	0.41793200

H	-3.94778900	-0.14205600	-2.85778100

H	-4.92637800	0.85212100	-1.78435300

H	-2.98119400	1.98004200	-0.85670400

H	-2.86850100	2.09066000	-2.61133000

H	-0.74377700	1.28838600	-3.62867100

H	0.01517800	-0.18977300	-3.02469800

H	-1.64537900	-0.23060700	-3.63762600

H	-1.75870300	-1.64465700	2.36450700

H	-3.50619200	-1.90136500	2.34162000

H	-3.32973100	-3.10262000	0.33722700

H	-1.58015900	-2.99337200	0.45463400

H	-3.10482900	-3.15670800	-1.98585200

H	-2.50956300	-1.83204400	-2.97374300

H	-1.36987900	-2.82658300	-2.05719500

H	-5.72825800	-1.19392100	-0.80336000

H	-1.05660300	0.98204800	3.09906300

H	-2.21949800	1.54522900	4.32230900

H	-2.02038400	-0.18828100	4.03792100

H	-4.08569200	2.35890900	1.23624100

H	-2.35444000	2.63989900	1.49631000

H	-3.51821100	2.95788300	2.80860000

H	-5.00903100	0.39911300	2.42972600

H	2.84702900	-3.33206200	-2.82870800

H	4.07899500	-4.15886200	-1.87805700

H	2.48325200	-4.93394600	-2.11291800

Energy = -2260.87932100 a.u. #Cluster: 4

C	-2.85585700	-0.15100900	1.32988800

O	4.92667400	-0.44852400	1.13609300

C	3.86027000	-1.30319800	1.59993900

C	2.69734000	-1.36507500	0.61978100

C	2.28244800	0.00654700	0.16192000

C	3.23644700	1.03939400	0.24499200

C	4.61459900	0.77689000	0.66626700

C	1.00565700	0.23144600	-0.34185800

C	0.66038700	1.54487500	-0.72988300

C	1.58939400	2.58973500	-0.63146700

C	2.89235500	2.35266800	-0.17435900

O	5.51813500	1.60737100	0.58703300

C	4.48468800	-2.65374100	1.89596300

O	3.76306300	3.36322200	-0.15833200

C	-0.00410100	-0.87990300	-0.49596400

C	-1.42401800	-0.33433700	-0.61865100

C	-1.44642900	0.82016700	-1.66516500

O	-0.56075800	1.87032600	-1.18928900

C	-2.50393300	-1.43595900	-0.83737700

C	-3.85477400	-0.67884300	-0.80962200

C	-3.94528100	0.44168800	-1.85276800

C	-2.82876600	1.47671100	-1.68310300

C	-0.95631600	0.46929100	-3.07401400

O	-1.71682500	0.31917800	0.63873700

O	-4.00974200	-0.05781300	0.47941900

C	-2.64072800	-1.61523300	1.69945300

C	-2.48155300	-2.39722700	0.37832000

C	-2.35766900	-2.28948000	-2.10338600

Cl	1.14091700	4.19310300	-1.13359300

O	-4.88069200	-1.62321800	-0.98341600

C	-3.12685000	0.84220900	2.49279500

C	-4.24391500	0.31660900	3.40625300

C	-1.85331500	1.12875100	3.28484100

O	-3.53217400	2.08924800	1.92667700

O	3.05589300	-2.15321200	-0.54699200

C	2.41489100	-3.33680800	-0.71777200

O	1.57420000	-3.77376800	0.03912600

C	2.89319700	-4.01144100	-1.97806200

H	3.46192900	-0.85717400	2.52147100

H	1.87760800	-1.88074400	1.12144900

H	5.26260800	-2.54957500	2.65641900

H	4.93652500	-3.06327300	0.98894900

H	3.72212200	-3.35098600	2.25672200

H	4.63820700	2.97619800	0.11201300

H	0.24012200	-1.49337600	-1.37034300

H	0.03442900	-1.55181200	0.36178400

H	-3.93646900	-0.00464900	-2.85256100

H	-4.91979200	0.93205600	-1.73414000

H	-2.94712900	2.00015000	-0.73255700

H	-2.86669400	2.22358800	-2.48257000

H	-0.02197600	-0.09823300	-3.06164700

H	-1.69453900	-0.09579500	-3.64314400

H	-0.77100400	1.40948500	-3.60105000

H	-1.75122900	-1.69469400	2.33121800

H	-3.49355600	-1.98730000	2.27008900

H	-3.30756000	-3.09920100	0.25127500

H	-1.55812300	-2.98304600	0.36382400

H	-1.36492500	-2.74689900	-2.14971400

H	-3.09645200	-3.09341300	-2.07118000

H	-2.52152400	-1.73769200	-3.02805000

H	-5.71979500	-1.16204300	-0.82274400

H	-4.51582400	1.10965800	4.10818800

H	-5.13105900	0.04917100	2.82290500

H	-3.93090800	-0.56095500	3.98112500

H	-1.08762600	1.54168400	2.62664500

H	-1.46756300	0.22088000	3.75995500

H	-2.07920600	1.85827100	4.06834100

H	-4.19449300	1.86096600	1.25269700

H	3.97093600	-4.19344000	-1.91762300

H	2.36272400	-4.95432600	-2.11183600

H	2.72344100	-3.35708600	-2.83905000

Energy = -2260.87794500 a.u. #Cluster: 5

C	-2.86346400	-0.11164300	1.33144800

O	4.91223400	-0.36763300	1.14596200

C	3.85916400	-1.23671600	1.61492200

C	2.70706200	-1.33781400	0.62570800

C	2.26487400	0.01810200	0.14694800

C	3.20008000	1.06923900	0.21324900

C	4.58026600	0.84097700	0.64848700

C	0.98434200	0.20990300	-0.36141900

C	0.61901000	1.50949400	-0.77998700

C	1.52854500	2.57246800	-0.69552100

C	2.83364600	2.36859400	-0.22914500

O	5.46796400	1.68760900	0.55925400

C	4.50840500	-2.56933000	1.93769700

O	3.68476900	3.39595200	-0.22522600

C	-0.01402000	-0.91816300	-0.47488300

C	-1.43724400	-0.38398600	-0.60769700

C	-1.46859600	0.72361200	-1.70494600

O	-0.60456100	1.80598100	-1.25448300

C	-2.52079700	-1.49292900	-0.77009000

C	-3.86736000	-0.72833300	-0.76200800

C	-3.97240100	0.31175600	-1.88317300

C	-2.85904600	1.35973600	-1.79083400

C	-0.94580200	0.32085400	-3.08839300

O	-1.70932300	0.32084200	0.62878400

O	-3.99862000	-0.01656000	0.47508300

C	-2.67996600	-1.56386200	1.76325800

C	-2.50192500	-2.40231400	0.48250800

C	-2.38293700	-2.39740400	-2.00047900

Cl	1.05044500	4.16013000	-1.22323800

O	-4.91062000	-1.67282300	-0.85240300

C	-3.05998700	0.89798200	2.48825500

C	-1.80262300	0.96923600	3.36480700

C	-3.40724200	2.28779200	1.94513100

O	-4.15730600	0.36836900	3.24351900

O	3.09644600	-2.12864300	-0.52867000

C	2.48741500	-3.33116400	-0.68981800

O	1.65466400	-3.78081400	0.06780300

C	2.98938200	-4.00611700	-1.94061500

H	3.44424400	-0.78420200	2.52593400

H	1.89506700	-1.86773000	1.12490600

H	3.75680800	-3.27616800	2.30263700

H	5.27730500	-2.43756000	2.70305600

H	4.97653200	-2.98375600	1.04119500

H	4.56588700	3.02929100	0.05484500

H	0.23099200	-1.56220200	-1.32638100

H	0.03174000	-1.55648800	0.40820500

H	-3.96911600	-0.20505400	-2.84834400

H	-4.94675300	0.80534300	-1.79177200

H	-3.00452200	1.95823200	-0.89127700

H	-2.89192700	2.04129700	-2.64733000

H	-0.00168600	-0.22710300	-3.03676800

H	-1.66462600	-0.28241300	-3.64298500

H	-0.77044800	1.24057100	-3.65368200

H	-1.80371300	-1.63757300	2.41228700

H	-3.55292300	-1.87255200	2.33991300

H	-3.31959100	-3.11678900	0.37482200

H	-1.57237600	-2.97877600	0.50200400

H	-1.40532800	-2.88913200	-2.01170200

H	-3.14916200	-3.17443400	-1.94774500

H	-2.51327900	-1.87851900	-2.94924000

H	-5.69276600	-1.24387600	-0.46883000

H	-1.95716200	1.70489800	4.16344800

H	-1.59541400	0.00376600	3.83401600

H	-0.93211700	1.27589600	2.78003200

H	-3.55003600	2.98397200	2.78107300

H	-4.32709500	2.24599600	1.35961900

H	-2.59844500	2.67306500	1.31836400

H	-4.33557500	0.99915900	3.95798900

H	4.07332100	-4.14614900	-1.88051700

H	2.49402500	-4.96985400	-2.05952900

H	2.79270800	-3.37142900	-2.81063900

**Table S9**. Important thermodynamic parameters and Boltzmann distributions of the optimized **6** at b3lyp/6-31g* level in gas phase.

| Conformations | Energy (a.u) | Δ G(kcal/mol) | % |
| --- | --- | --- | --- |
| 1 | -2033.026175 | 0 | 43.99% |
| 2 | -2033.025401 | 0.49 | 19.38% |
| 3 | -2033.025335 | 0.53 | 18.07% |
| 4 | -2033.02488 | 0.81 | 11.16% |
| 5 | -2033.024493 | 1.06 | 7.41% |

**Table S10**. The coordinates for the lowest energy conformers of **6**.

Energy = -2033.02617500 a.u. #Cluster: 1

C	-2.58274500	-0.63879700	1.16748900

O	5.04798500	-1.81798300	0.08448500

C	3.87012800	-2.65609500	0.18728000

C	2.77349600	-2.14343600	-0.73520900

C	2.48017600	-0.69018700	-0.45482900

C	3.55903100	0.09303600	0.00444800

C	4.89579100	-0.48184600	0.18532700

C	1.22176900	-0.13075800	-0.66231100

C	1.04885200	1.24836600	-0.40332400

C	2.11086800	2.03621500	0.05754900

C	3.38169400	1.48133800	0.25006300

O	5.89994300	0.19326900	0.40853000

C	4.32553800	-4.06903300	-0.12520000

O	4.37823600	2.27413600	0.64718900

C	0.04123800	-0.95780400	-1.12351500

C	-1.28053400	-0.23763700	-0.85123300

C	-1.17014500	1.23569400	-1.34957900

O	-0.13235800	1.87813500	-0.56155300

C	-2.54856400	-0.99832200	-1.34697500

C	-3.74501800	-0.13394400	-0.86963400

C	-3.71461000	1.29204200	-1.42815500

C	-2.43768600	2.02787500	-1.01380500

C	-0.76654300	1.42058100	-2.81703000

O	-1.37877300	-0.13687900	0.58994600

O	-3.69836800	-0.00179000	0.56296200

C	-2.65236000	-2.14180300	0.91897400

C	-2.63593100	-2.35623000	-0.60707400

C	-2.61708800	-1.27895200	-2.85294800

Cl	1.86302700	3.73310900	0.35833200

O	-4.92647700	-0.80537300	-1.23295300

C	-2.50855700	-0.21499200	2.65717900

C	-2.46624100	1.31269700	2.78577300

C	-3.66727300	-0.79532900	3.46282200

O	-1.31182800	-0.78487200	3.19548500

H	3.51997600	-2.59173700	1.22645100

H	3.09216200	-2.27062000	-1.78039200

H	1.88448700	-2.76639800	-0.60024800

H	5.13651200	-4.36523400	0.54582100

H	4.68759500	-4.13511200	-1.15640300

H	3.49345700	-4.76968000	0.00072100

H	5.20027500	1.71365000	0.64658500

H	0.13164500	-1.20933300	-2.18689000

H	0.02047400	-1.90489900	-0.57949000

H	-3.83300200	1.25484100	-2.51571700

H	-4.58558400	1.82765200	-1.03136500

H	-2.45182400	2.18954600	0.06394200

H	-2.38292900	3.01133600	-1.49193400

H	-1.58816500	1.21071200	-3.50226300

H	-0.47761900	2.46719500	-2.94827800

H	0.08671900	0.79945600	-3.09973400

H	-1.80664600	-2.61358000	1.42377900

H	-3.57069200	-2.53957900	1.35600900

H	-3.55327400	-2.84759800	-0.93481300

H	-1.80386600	-2.99481100	-0.91800000

H	-2.71528600	-0.38356500	-3.46517500

H	-1.72734900	-1.82021900	-3.18837300

H	-3.48848100	-1.90626000	-3.05442500

H	-5.65683300	-0.34485900	-0.78881700

H	-2.33591800	1.57430700	3.83998900

H	-1.62914700	1.72564400	2.21612300

H	-3.39126700	1.76639200	2.41901500

H	-3.62478900	-0.41417300	4.48729500

H	-3.60577200	-1.88576800	3.50674300

H	-4.62422100	-0.50803400	3.01824700

H	-0.59070000	-0.45735600	2.63072200

Energy = -2033.02540100 a.u. #Cluster: 2

C	-2.55338700	-0.67651000	1.14682600

O	5.05759500	-1.82559900	0.05384300

C	3.87743700	-2.66109400	0.14753900

C	2.77809900	-2.12933700	-0.76075300

C	2.49177400	-0.67983200	-0.45555200

C	3.57558400	0.09203700	0.01140100

C	4.91049100	-0.49036900	0.17752600

C	1.23507400	-0.11273400	-0.64910700

C	1.06792900	1.26249000	-0.36893700

C	2.13489100	2.03944600	0.09896400

C	3.40400400	1.47698100	0.27918000

O	5.91830100	0.17657900	0.40907900

C	4.32661700	-4.07003400	-0.19129200

O	4.40522800	2.26003300	0.68459200

C	0.05085000	-0.92807400	-1.11957900

C	-1.26892400	-0.20525200	-0.84716000

C	-1.15237600	1.27612700	-1.31381600

O	-0.11062600	1.89912500	-0.51604400

C	-2.53241200	-0.95529800	-1.37708200

C	-3.73444200	-0.11189200	-0.88414600

C	-3.69738400	1.33772800	-1.37931000

C	-2.41763300	2.05781600	-0.94635900

C	-0.75386000	1.49467300	-2.77745800

O	-1.38140400	-0.11394800	0.59707700

O	-3.70589500	-0.06089800	0.55266100

C	-2.58456900	-2.17241900	0.85094500

C	-2.63290900	-2.33506800	-0.68020000

C	-2.58913200	-1.18904300	-2.89190900

Cl	1.89532000	3.73322100	0.42414300

O	-4.91035500	-0.76151200	-1.30015300

C	-2.60312300	-0.29748300	2.64835500

C	-1.48350100	-0.98160500	3.42905300

C	-2.52849200	1.22406100	2.82707500

O	-3.83469400	-0.80056500	3.17477800

H	3.53204900	-2.61340300	1.18926200

H	3.09081500	-2.24114500	-1.80950700

H	1.88727600	-2.75089200	-0.63107500

H	5.13995100	-4.38038200	0.47043000

H	4.68350200	-4.11943600	-1.22521700

H	3.49293100	-4.77023600	-0.07361200

H	5.22487600	1.69635900	0.67252800

H	0.14351900	-1.16981400	-2.18509800

H	0.02284700	-1.88105200	-0.58560200

H	-3.82038400	1.34423800	-2.46710200

H	-4.56583300	1.85950400	-0.95820600

H	-2.42159500	2.18219900	0.13639700

H	-2.36579500	3.05673800	-1.39148700

H	0.09031400	0.86972400	-3.07907800

H	-1.58025000	1.31329100	-3.46479700

H	-0.45151900	2.54043700	-2.88254600

H	-1.68818300	-2.63352100	1.27278300

H	-3.45281200	-2.61523700	1.34248100

H	-3.57647700	-2.78631000	-0.99112400

H	-1.83197900	-2.98642600	-1.04250200

H	-1.68333000	-1.69193300	-3.24336500

H	-3.44091500	-1.83531800	-3.11574200

H	-2.71469500	-0.27710300	-3.47410400

H	-5.65523300	-0.28251200	-0.90254100

H	-1.49799000	-0.63298600	4.46573300

H	-1.61823200	-2.06636100	3.43778400

H	-0.51210100	-0.74149900	2.98849800

H	-1.58366100	1.62110200	2.44667000

H	-2.61519500	1.46091300	3.89154900

H	-3.34754400	1.71561700	2.29421000

H	-4.53364000	-0.42693800	2.61205300

Energy = -2033.02533500 a.u. #Cluster: 3

C	-2.62601400	-0.54136600	1.20540900

O	5.09661700	-1.76933900	0.20740700

C	3.93326000	-2.61559300	0.38123300

C	2.81848900	-2.18398700	-0.56056800

C	2.50650900	-0.71931400	-0.37778700

C	3.57800800	0.10999000	0.01257900

C	4.92530400	-0.43147200	0.21680800

C	1.23795300	-0.19430700	-0.60981700

C	1.04849900	1.19709700	-0.45086500

C	2.10188300	2.03139900	-0.05876000

C	3.38248200	1.50950600	0.16091900

O	5.92165300	0.27156200	0.37999200

C	4.40749100	-4.03967400	0.16042600

O	4.37087000	2.34305300	0.48888200

C	0.06669300	-1.06915100	-1.00129700

C	-1.26633700	-0.34397600	-0.80625800

C	-1.15606000	1.08969600	-1.41669200

O	-0.14557400	1.79537200	-0.64554700

C	-2.51600900	-1.15922500	-1.25930800

C	-3.72517500	-0.25461100	-0.91716300

C	-3.68171700	1.10957600	-1.61146300

C	-2.43865600	1.89238600	-1.18388800

C	-0.71443100	1.16485200	-2.88300400

O	-1.39010500	-0.13316700	0.61931100

O	-3.71422100	0.02222000	0.48952900

C	-2.70883600	-2.06143600	1.11971200

C	-2.63733600	-2.43055900	-0.37933700

C	-2.52832100	-1.59965600	-2.72708400

Cl	1.83174200	3.74180700	0.11841200

O	-4.89653600	-0.96404300	-1.24303700

C	-2.61232900	0.10392300	2.61728900

C	-3.98259900	0.01278200	3.28245800

C	-1.52545000	-0.53643700	3.49368600

O	-2.33424200	1.49534200	2.46257400

H	3.59350000	-2.48522000	1.41773400

H	3.12713200	-2.37849000	-1.59843700

H	1.94031400	-2.80866600	-0.37209700

H	4.75987900	-4.17028500	-0.86794600

H	3.58766900	-4.74244500	0.34257100

H	5.22988700	-4.27744200	0.84069400

H	5.20145000	1.79640000	0.51801200

H	0.16610400	-1.40930900	-2.03901500

H	0.05190700	-1.96659600	-0.37775200

H	-3.73399900	0.96835100	-2.69598800

H	-4.57914000	1.66652100	-1.31676400

H	-2.51766100	2.11513000	-0.11866000

H	-2.36819000	2.84488700	-1.71899500

H	0.14161100	0.52204700	-3.10064500

H	-1.52292700	0.90685200	-3.56786700

H	-0.41985200	2.19854900	-3.08511300

H	-1.88111500	-2.50380100	1.67838700

H	-3.64436900	-2.40427600	1.56950200

H	-3.54065400	-2.95803200	-0.68924100

H	-1.79252500	-3.09427500	-0.58516400

H	-1.67474300	-2.24938400	-2.94367900

H	-3.44128600	-2.16920600	-2.91584600

H	-2.50701100	-0.77186700	-3.43468800

H	-5.63817500	-0.46146900	-0.86864400

H	-3.94201400	0.51608200	4.25296000

H	-4.73509300	0.50310600	2.66275500

H	-4.27601600	-1.02925400	3.44479000

H	-0.55876900	-0.52485800	2.98062600

H	-1.76563000	-1.57071400	3.76199300

H	-1.43734300	0.04657000	4.41455500

H	-1.55406200	1.54973700	1.88269900

Energy = -2033.02488000 a.u. #Cluster: 4

C	-2.70421400	0.15432000	1.24681500

O	5.03570000	-1.51960500	1.35717900

C	4.16114600	-2.57190600	0.87383500

C	2.69880700	-2.15189200	0.99835900

C	2.46391900	-0.82124600	0.32769600

C	3.53299600	0.09553100	0.34567300

C	4.81997300	-0.25001500	0.95662100

C	1.24051100	-0.49020600	-0.24497400

C	1.09722400	0.78494200	-0.83655200

C	2.14337900	1.71552300	-0.80401000

C	3.36611500	1.39685000	-0.19970300

O	5.72137400	0.56709000	1.14086600

C	4.57036400	-2.96642900	-0.54182300

O	4.33117400	2.31725000	-0.16896200

C	0.06147500	-1.43815500	-0.20166200

C	-1.24716000	-0.70640700	-0.50432800

C	-1.04491800	0.19018100	-1.76644400

O	-0.04429600	1.18829700	-1.42879200

C	-2.50604800	-1.62457900	-0.55449900

C	-3.69781400	-0.65813300	-0.77976400

C	-3.57255900	0.15572100	-2.07110800

C	-2.30283300	1.01075400	-2.06798200

C	-0.52879700	-0.52468800	-3.02113500

O	-1.44017700	0.22287700	0.58777900

O	-3.74496100	0.30159000	0.29206900

C	-2.82360000	-1.20360200	1.92997200

C	-2.69758200	-2.28077800	0.83528500

C	-2.48031600	-2.74432900	-1.60211400

Cl	1.92239300	3.29097800	-1.51131800

O	-4.87687000	-1.42560000	-0.76496900

C	-2.71754300	1.37940800	2.19735800

C	-2.61169400	2.68639000	1.40155800

C	-3.95775800	1.38389100	3.08641500

O	-1.59242900	1.24534800	3.07036400

H	4.36411000	-3.40013700	1.55712400

H	2.07112000	-2.93564000	0.56395100

H	2.43051100	-2.08260300	2.06201100

H	4.01588800	-3.85658000	-0.85856700

H	5.63958200	-3.19283400	-0.57350100

H	4.36333000	-2.16046200	-1.25245500

H	5.08504600	1.91537700	0.34123300

H	0.20175500	-2.27025000	-0.90230200

H	-0.01832100	-1.87516100	0.79648500

H	-3.60906200	-0.52288400	-2.92908900

H	-4.44959900	0.81010600	-2.14431500

H	-2.39115700	1.77746100	-1.29833200

H	-2.17547200	1.52313400	-3.02719000

H	0.32611900	-1.17244800	-2.81289200

H	-1.30216700	-1.12038000	-3.50683400

H	-0.20300100	0.24483600	-3.72672000

H	-2.04348000	-1.27663400	2.69051400

H	-3.79141900	-1.27277000	2.43124900

H	-3.60337900	-2.88724500	0.78848500

H	-1.86646100	-2.96424000	1.03281400

H	-1.59818200	-3.37824300	-1.47077000

H	-3.36697300	-3.36868400	-1.47072500

H	-2.48642700	-2.38659000	-2.63081600

H	-5.61711600	-0.79809100	-0.73404300

H	-2.53923900	3.52161600	2.10425300

H	-1.72029000	2.68284600	0.76809400

H	-3.48818700	2.83385100	0.76431200

H	-3.97026300	2.29724000	3.68823000

H	-3.95165400	0.53123600	3.77032500

H	-4.86561500	1.34886900	2.47782200

H	-0.81522700	1.18383100	2.48883500

Energy = -2033.02449300 a.u. #Cluster: 5

C	-2.70841200	0.20439100	1.24584600

O	5.06414500	-1.45209000	1.38400500

C	4.19389100	-2.52619700	0.94267900

C	2.72942500	-2.11352200	1.06702200

C	2.47801400	-0.80716700	0.35589300

C	3.54007800	0.11775700	0.33374900

C	4.83500000	-0.19779500	0.94406500

C	1.24669300	-0.50355800	-0.21452000

C	1.08861600	0.75027800	-0.84578500

C	2.12735900	1.68895400	-0.85493900

C	3.35807300	1.39932100	-0.25175100

O	5.73143600	0.63161900	1.09399100

C	4.59374900	-2.96270200	-0.46325000

O	4.31583800	2.32773300	-0.26014900

C	0.07457400	-1.45704100	-0.12823100

C	-1.24173400	-0.74814500	-0.45064000

C	-1.05254200	0.10481000	-1.74599400

O	-0.06473700	1.12662300	-1.43657700

C	-2.49193700	-1.67987200	-0.44996300

C	-3.68782100	-0.74127600	-0.73980400

C	-3.56651000	0.00729600	-2.07014600

C	-2.32094200	0.89588400	-2.07852000

C	-0.52557000	-0.64714600	-2.97359100

O	-1.43337400	0.22132400	0.60508900

O	-3.74233900	0.26882100	0.27527000

C	-2.83011500	-1.10683900	2.01463800

C	-2.69803100	-2.24679600	0.97907000

C	-2.44523600	-2.86242300	-1.42416200

Cl	1.88663700	3.23890900	-1.60975500

O	-4.86258800	-1.51564900	-0.68833500

C	-2.74693200	1.52800400	2.05578300

C	-4.15086700	1.81879900	2.57804500

C	-1.72297900	1.49259800	3.19987900

O	-2.42315600	2.59689000	1.16646900

H	4.40965700	-3.33044100	1.65031000

H	2.10404100	-2.91566500	0.66395000

H	2.47033100	-2.01183500	2.13038800

H	5.66446100	-3.18160100	-0.49728400

H	4.37398200	-2.18149600	-1.19733200

H	4.04384300	-3.86689800	-0.74647200

H	5.07754400	1.94933900	0.25601500

H	0.21614700	-2.31203000	-0.80025700

H	0.00405700	-1.85978800	0.88532500

H	-3.57104500	-0.71225400	-2.89514300

H	-4.45839300	0.63442300	-2.18612700

H	-2.44662400	1.67578000	-1.32585000

H	-2.19343800	1.38764600	-3.04826700

H	0.33790200	-1.27551300	-2.74304000

H	-1.29169800	-1.27088200	-3.43562900

H	-0.21168900	0.10001300	-3.70801400

H	-2.04253900	-1.16206000	2.76917500

H	-3.79508200	-1.14577800	2.52699400

H	-3.60374400	-2.85461300	0.95991100

H	-1.86868100	-2.91541500	1.22768700

H	-1.58681300	-3.50654200	-1.21069100

H	-3.35284900	-3.45720900	-1.29899400

H	-2.39113300	-2.56687300	-2.47136100

H	-5.60600800	-0.89110900	-0.69547100

H	-4.85691600	1.87156200	1.74771900

H	-4.48108200	1.04705600	3.28056400

H	-4.14505600	2.78102600	3.09837800

H	-1.67019100	2.48934600	3.64646000

H	-0.73005900	1.22830300	2.82269200

H	-1.99979600	0.77860100	3.98276300

H	-1.60884700	2.32561600	0.70708700

**Table S11**. Important thermodynamic parameters and Boltzmann distributions of the optimized **7** at b3lyp/6-31g* level in gas phase.

| Conformations | Energy (a.u) | Δ G(kcal/mol) | % |
| --- | --- | --- | --- |
| 1 | -2032.993008 | 0 | 56.24% |
| 2 | -2032.992302 | 0.44 | 26.63% |
| 3 | -2032.99182 | 0.75 | 15.98% |
| 4 | -2032.988834 | 2.62 | 0.68% |
| 5 | -2032.988497 | 2.83 | 0.47% |

**Table S12**. The coordinates for the lowest energy conformers of **7**.

Energy = -2032.99300800 a.u. #Cluster: 1

C	2.34376500	-0.62862700	-1.57204500

O	-4.87163500	-1.87902500	-0.48928000

C	-4.01683500	-2.55809600	0.46691000

C	-2.55013800	-2.18384100	0.26458700

C	-2.37840300	-0.68317100	0.20176200

C	-3.46764800	0.08229100	-0.26138900

C	-4.72718800	-0.54807300	-0.66317600

C	-1.15739600	-0.09681100	0.50858700

C	-1.02919500	1.30196000	0.37615300

C	-2.10529300	2.08036500	-0.07402200

C	-3.32683900	1.48841200	-0.41840900

O	-5.65162400	0.06344500	-1.19722900

C	-4.52165900	-2.29217000	1.88096700

O	-4.31196300	2.26140700	-0.88037200

C	0.04572200	-0.92603400	0.88686300

C	1.34111600	-0.15490400	0.60905800

C	1.22764500	1.24944400	1.26347400

O	0.11669200	1.95545000	0.63261000

C	2.63446000	-0.95027300	0.96417400

C	3.81735400	-0.08970700	0.40449000

C	3.76716300	1.32325800	1.03482500

C	2.44938400	2.09423200	0.89955700

C	0.93312800	1.27892600	2.76559600

O	1.38769200	0.12873300	-0.81242700

C	3.77066900	-0.11811800	-1.19296800

C	2.17225300	-2.10659100	-1.25772200

C	2.57908300	-2.30711900	0.21195800

C	2.82833700	-1.28066100	2.45241100

Cl	-1.91231600	3.80176600	-0.24699000

O	2.02173400	-0.42627400	-2.91661800

C	4.83395500	-1.07423900	-1.77590800

C	4.03049000	1.24459200	-1.86128400

O	5.00890600	-0.71982600	0.89221600

O	-1.98243000	-2.82216800	-0.88239200

H	-4.13366800	-3.61361700	0.21165200

H	-1.99232300	-2.59450100	1.11084100

H	-5.58606000	-2.53243900	1.94725400

H	-4.38344100	-1.24271300	2.15948200

H	-3.97891100	-2.91454800	2.60053600

H	-5.05813900	1.65018700	-1.12250900

H	0.00682500	-1.22006100	1.94299700

H	0.03655600	-1.84297000	0.30050600

H	4.00835400	1.19230600	2.09420300

H	4.57924200	1.93281200	0.62290300

H	2.47750800	2.98719800	1.53389800

H	2.29559600	2.44024100	-0.12041500

H	0.08763600	0.64127600	3.03508800

H	1.79460300	0.97948400	3.36171000

H	0.67484300	2.30801800	3.03067200

H	1.12493600	-2.36059700	-1.44301100

H	2.77592100	-2.71049600	-1.93824200

H	3.56940200	-2.76190300	0.29324100

H	1.88707700	-2.98193900	0.72487200

H	1.90933400	-1.68507000	2.88753100

H	3.60760400	-2.04001600	2.54616000

H	3.14457300	-0.43053900	3.05559800

H	1.84943500	0.52352700	-3.02453900

H	4.87461300	-2.03930300	-1.27216600

H	5.83355700	-0.62957100	-1.71090000

H	4.61897500	-1.23630100	-2.83679200

H	4.97089100	1.68370100	-1.51313100

H	4.12614000	1.10450000	-2.94195900

H	3.23669400	1.97259200	-1.68774100

H	5.76647200	-0.21390300	0.56016900

H	-2.31742200	-2.35958400	-1.66784100

Energy = -2032.99230200 a.u. #Cluster: 2

C	2.33799400	-0.64126500	-1.56675700

O	-4.86704400	-1.88460200	-0.48797900

C	-4.01599700	-2.55864300	0.47478700

C	-2.54872900	-2.18233200	0.27988600

C	-2.37912200	-0.68173300	0.21015400

C	-3.46764500	0.08003900	-0.26050900

C	-4.72450100	-0.55394800	-0.66533500

C	-1.15987400	-0.09213700	0.51804800

C	-1.03253700	1.30570100	0.37755900

C	-2.10844900	2.08092900	-0.07825900

C	-3.32831200	1.48569500	-0.42315200

O	-5.64781500	0.05420100	-1.20499500

C	-4.52919000	-2.28893000	1.88516400

O	-4.31299100	2.25532900	-0.89132600

C	0.04373400	-0.91666700	0.90421600

C	1.33870300	-0.14856900	0.61397900

C	1.22972800	1.26176700	1.25635500

O	0.11230100	1.96216300	0.63206400

C	2.63401300	-0.94075800	0.96657300

C	3.82155000	-0.08313500	0.38847900

C	3.76795200	1.33739900	1.00124900

C	2.44691300	2.10210900	0.86852200

C	0.95129400	1.30775400	2.76138900

O	1.38276500	0.12386700	-0.80776100

C	3.77014400	-0.13636800	-1.19858300

C	2.15922400	-2.11562200	-1.23920900

C	2.57653000	-2.30394900	0.22889300

C	2.83931000	-1.26016300	2.45514700

Cl	-1.91706800	3.80154900	-0.25916500

O	2.01056700	-0.44940300	-2.90972000

C	4.82726000	-1.10470300	-1.77502500

C	4.04424500	1.21699200	-1.87936700

O	5.07841000	-0.65818500	0.76450000

O	-1.97169300	-2.82604800	-0.85929500

H	-4.12918200	-3.61527600	0.22253500

H	-1.99608900	-2.58713400	1.13243800

H	-4.39432800	-1.23827600	2.16088700

H	-3.98952300	-2.90802900	2.60988200

H	-5.59355400	-2.53071900	1.94623800

H	-5.05735900	1.64207400	-1.13390600

H	0.00661100	-1.19777300	1.96430700

H	0.03429700	-1.84072200	0.32935200

H	4.00283400	1.23301200	2.06966300

H	4.58256600	1.93354200	0.58258300

H	2.47905400	3.00641200	1.48639200

H	2.28530000	2.42816600	-0.15667400

H	1.82237300	1.02787700	3.35382000

H	0.68423900	2.33725200	3.01579700

H	0.11629600	0.66426800	3.04938200

H	1.10913900	-2.36594500	-1.41360700

H	2.75559700	-2.72871800	-1.91744800

H	3.57141300	-2.75089400	0.30413800

H	1.89260500	-2.97993400	0.75162400

H	3.61725000	-2.02401300	2.55086300

H	3.14364600	-0.40433200	3.06023500

H	1.92924700	-1.66970200	2.90257000

H	1.88862300	0.50561400	-3.03790900

H	4.84359200	-2.07624400	-1.28208100

H	5.82637000	-0.67587800	-1.67100200

H	4.62006100	-1.25397600	-2.83958500

H	3.26226800	1.95930000	-1.70964100

H	4.99370700	1.63728600	-1.53616700

H	4.13360600	1.06573000	-2.95916600

H	5.20396700	-0.49018600	1.71099600

H	-2.29589700	-2.36446300	-1.64984600

Energy = -2032.99182000 a.u. #Cluster: 3

C	2.39059500	-0.62010500	-1.56693900

O	-4.88672600	-1.88724500	-0.46298500

C	-4.01894000	-2.55818300	0.48806500

C	-2.55763700	-2.18828600	0.24767400

C	-2.38584700	-0.68734400	0.18633700

C	-3.48062200	0.07650800	-0.26698400

C	-4.74513000	-0.55549800	-0.64770700

C	-1.16431800	-0.09854200	0.48494900

C	-1.03964100	1.30120100	0.35277600

C	-2.12040500	2.07755700	-0.09110600

C	-3.34353500	1.48290000	-0.42534600

O	-5.67718900	0.05098300	-1.17327200

C	-4.49646500	-2.27548500	1.90793500

O	-4.33452700	2.25393100	-0.87773600

C	0.04099900	-0.92281000	0.86724900

C	1.33829100	-0.15099000	0.59637300

C	1.21824200	1.25791100	1.24038600

O	0.10419100	1.95634500	0.60628800

C	2.62971600	-0.94014100	0.96943900

C	3.82648700	-0.08060800	0.41025100

C	3.75786400	1.33977500	1.02221000

C	2.43706300	2.09997400	0.86365300

C	0.92385200	1.30159200	2.74251700

O	1.40722600	0.12770900	-0.82293900

C	3.81174900	-0.12758300	-1.18011000

C	2.19739500	-2.10045900	-1.24365100

C	2.58323000	-2.30200800	0.23094900

C	2.81383600	-1.26101900	2.46049400

Cl	-1.93371900	3.79893700	-0.26149800

O	2.15462900	-0.33247200	-2.91122100

C	4.87866200	-1.10174800	-1.73118000

C	4.10314900	1.22763300	-1.84934800

O	5.07534000	-0.65586900	0.81080000

O	-2.03047500	-2.81879000	-0.92385300

H	-4.14414100	-3.61627800	0.24722500

H	-1.97341800	-2.60716100	1.07101400

H	-3.93899900	-2.88898500	2.62390900

H	-5.55894700	-2.51675800	1.99745400

H	-4.35474900	-1.22285800	2.17174200

H	-5.08302700	1.64281400	-1.11064000

H	-0.00301400	-1.21546000	1.92397400

H	0.03784700	-1.84216300	0.28425600

H	3.97420400	1.23691600	2.09494800

H	4.57674100	1.93818400	0.61627600

H	2.45707100	3.00850800	1.47589600

H	2.29117100	2.41503800	-0.16714900

H	0.09290400	0.64938400	3.02316900

H	1.79193300	1.03165000	3.34376700

H	0.64382000	2.32847700	2.99349500

H	1.14780000	-2.35429100	-1.43204900

H	2.80460000	-2.71219900	-1.91365100

H	3.57578800	-2.75121600	0.32047100

H	1.88862100	-2.97846800	0.73893900

H	1.89781800	-1.67124400	2.89509500

H	3.59086400	-2.02439900	2.56594800

H	3.10987600	-0.40601200	3.07045900

H	1.22302900	-0.54845300	-3.08138000

H	5.87535900	-0.67869500	-1.59006300

H	4.70764600	-1.23876300	-2.80350600

H	4.87453200	-2.07827700	-1.24706100

H	3.30664300	1.95685400	-1.70757600

H	5.04323700	1.64454400	-1.47509300

H	4.20926900	1.07914900	-2.92577400

H	5.19203600	-0.46958200	1.75481700

H	-2.47460000	-2.41439300	-1.68740600

Energy = -2032.98883400 a.u. #Cluster: 4

C	2.25044200	-1.03349900	-1.34321400

O	-4.95411100	-1.76225300	0.20997300

C	-3.81133400	-2.58184500	-0.11302900

C	-2.51721400	-2.06115600	0.52560600

C	-2.37714300	-0.55840200	0.35250700

C	-3.52107900	0.16365400	-0.06229800

C	-4.85452600	-0.44245800	-0.04845900

C	-1.15724100	0.08618500	0.55242800

C	-1.04918300	1.44465600	0.17118200

C	-2.16181600	2.15785700	-0.29017000

C	-3.42061400	1.54970100	-0.35842500

O	-5.89508300	0.19431300	-0.20629600

C	-4.14867700	-3.99736200	0.31078400

O	-4.47400800	2.29050600	-0.70678600

C	0.05110600	-0.58323600	1.17141700

C	1.34284700	0.05657800	0.65347400

C	1.26990100	1.58774500	0.90223200

O	0.11402000	2.11804500	0.19063400

C	2.65321900	-0.61912900	1.16341500

C	3.80767300	0.03458200	0.32736100

C	3.78623600	1.56911100	0.52459200

C	2.45982600	2.28154600	0.23919100

C	1.08872300	2.04170000	2.35476500

O	1.32794900	-0.07807500	-0.79325500

C	3.68913100	-0.44852200	-1.19372100

C	2.11038400	-2.35898900	-0.60979800

C	2.57659200	-2.13256900	0.83597700

C	2.91097300	-0.51404200	2.67515600

Cl	-1.99746800	3.83498600	-0.72620300

O	1.85738900	-1.23044600	-2.67065300

C	4.73412800	-1.53481400	-1.52709800

C	3.90477500	0.66613800	-2.23544500

O	5.02117800	-0.44450100	0.92056700

O	-1.44438700	-2.79154600	-0.08541500

H	-3.67146400	-2.53462500	-1.20220500

H	-2.48936000	-2.33653400	1.58697000

H	-5.03676700	-4.35420600	-0.21767800

H	-4.34828600	-4.03161100	1.38713500

H	-3.30578200	-4.65519600	0.08471000

H	-5.27308300	1.70579300	-0.60852300

H	0.00118200	-0.49245200	2.26361800

H	0.03906500	-1.64531500	0.95112200

H	4.08128100	1.74719500	1.56331800

H	4.57479100	2.02395800	-0.08535800

H	2.51549100	3.31939400	0.58562600

H	2.24765600	2.31845700	-0.82724100

H	0.80609100	3.09779800	2.33101400

H	0.29497800	1.49527200	2.86995900

H	2.00497900	1.94886900	2.93672800

H	1.06334600	-2.66420000	-0.66155100

H	2.69389000	-3.13047500	-1.11625900

H	3.57260900	-2.54959400	1.00435800

H	1.90924100	-2.62875100	1.54695700

H	2.00054200	-0.73105000	3.24214100

H	3.66660800	-1.25056600	2.95680400

H	3.28721300	0.45718600	2.99282100

H	1.72315600	-0.35013100	-3.05868700

H	5.73090900	-1.09249700	-1.63639800

H	4.47498600	-1.99342000	-2.48651100

H	4.80738700	-2.31447800	-0.77015500

H	3.11088200	1.41445300	-2.24023900

H	4.85451700	1.18514700	-2.07226900

H	3.95538100	0.22253300	-3.23449500

H	5.76157300	-0.05142200	0.43316600

H	-1.22239900	-2.33322700	-0.91416500

Energy = -2032.98849700 a.u. #Cluster: 5

C	2.25168000	-1.03943100	-1.33745300

O	-4.95348500	-1.76695800	0.20969900

C	-3.80822200	-2.58442500	-0.11013400

C	-2.51733500	-2.06066000	0.53242900

C	-2.37897000	-0.55807300	0.35614200

C	-3.52349100	0.16146900	-0.06115400

C	-4.85595700	-0.44706800	-0.04866800

C	-1.16021400	0.08889500	0.55556700

C	-1.05378400	1.44620700	0.17065500

C	-2.16712500	2.15735900	-0.29192200

C	-3.42504200	1.54719500	-0.35946800

O	-5.89734400	0.18793200	-0.20781100

C	-4.14400200	-4.00050900	0.31298200

O	-4.47929100	2.28587000	-0.70942600

C	0.04905400	-0.57555200	1.17778500

C	1.34044700	0.06124700	0.65405300

C	1.26900400	1.59402400	0.89378500

O	0.10863000	2.12139500	0.18851300

C	2.65229400	-0.61149900	1.16189600

C	3.81210200	0.03808800	0.31400300

C	3.78418800	1.57429100	0.49721300

C	2.45417700	2.28053000	0.21532300

C	1.09931500	2.06001000	2.34434900

O	1.32684200	-0.08029600	-0.79009600

C	3.69372900	-0.46074600	-1.19226300

C	2.10604400	-2.36017300	-0.59631500

C	2.57802700	-2.12605900	0.84609600

C	2.91769700	-0.49952000	2.67129000

Cl	-2.00495500	3.83381300	-0.73090900

O	1.85675100	-1.24232100	-2.66178700

C	4.73464500	-1.55430100	-1.51873000

C	3.92341700	0.64516400	-2.23951900

O	5.08439100	-0.42224300	0.78364800

O	-1.44086000	-2.79090100	-0.07167600

H	-3.66533900	-2.53706500	-1.19890400

H	-2.49399800	-2.33332500	1.59464500

H	-4.34657500	-4.03502300	1.38878000

H	-3.29929100	-4.65680100	0.08928300

H	-5.02995200	-4.35904400	-0.21790500

H	-5.27761100	1.70009700	-0.61126200

H	-0.00027300	-0.47684000	2.26968700

H	0.03773700	-1.63922600	0.96512700

H	4.07153900	1.77860900	1.53786200

H	4.57557000	2.01728000	-0.11231100

H	2.51053400	3.32339300	0.54635300

H	2.23701700	2.30060900	-0.85041500

H	0.32158600	1.50680500	2.87657000

H	2.02453000	1.99081100	2.91612500

H	0.79985700	3.11121500	2.31333600

H	1.05744400	-2.66066500	-0.64198200

H	2.68511800	-3.13718800	-1.09864900

H	3.57877900	-2.53563700	1.00782500

H	1.91789400	-2.62380000	1.56310500

H	2.01802400	-0.73119600	3.24849200

H	3.68024800	-1.23184700	2.95410700

H	3.26947900	0.48130400	2.99494800

H	1.75913300	-0.36477000	-3.06652200

H	5.73176900	-1.11384800	-1.58744400

H	4.48258700	-2.00008800	-2.48633500

H	4.78581200	-2.34455100	-0.77081900

H	3.14121100	1.40621000	-2.25009600

H	4.88249400	1.14330700	-2.07346000

H	3.96804700	0.19467500	-3.23582200

H	5.24277400	-0.01060700	1.64695400

H	-1.21704800	-2.33621100	-0.90199900
